# Mycofabricated biosilver nanoparticles interrupt *Pseudomonas aeruginosa* quorum sensing systems

**DOI:** 10.1038/srep13719

**Published:** 2015-09-08

**Authors:** Braj R. Singh, Brahma N. Singh, Akanksha Singh, Wasi Khan, Alim H. Naqvi, Harikesh B. Singh

**Affiliations:** 1 Centre of Excellence in Materials Science (Nanomaterials), Z.H. College of Engineering & Technology, Aligarh Muslim University, Aligarh-202002, India; 2 DSIR-recognized Research and Development Division, Sowbhagya Biotech Private Limited, Cherlapally, Hyderabad-500051, India; 3 Pharmacognosy & Ethnopharmacology Division, CSIR-National Botanical Research Institute, Lucknow-226001, India; 4 Department of Mycology and Plant Pathology, Institute of Agricultural Sciences, Banaras Hindu University, Varanasi-221005, India

## Abstract

Quorum sensing (QS) is a chemical communication process that *Pseudomonas aeruginosa* uses to regulate virulence and biofilm formation. Disabling of QS is an emerging approach for combating its pathogenicity. Silver nanoparticles (AgNPs) have been widely applied as antimicrobial agents against human pathogenic bacteria and fungi, but not for the attenuation of bacterial QS. Here we mycofabricated AgNPs (mfAgNPs) using metabolites of soil fungus *Rhizopus arrhizus* BRS-07 and tested their effect on QS-regulated virulence and biofilm formation of *P*. *aeruginosa*. Transcriptional studies demonstrated that mfAgNPs reduced the levels of LasIR-RhlIR. Treatment of mfAgNPs inhibited biofilm formation, production of several virulence factors (e.g. LasA protease, LasB elastrase, pyocyanin, pyoverdin, pyochelin, rhamnolipid, and alginate) and reduced AHLs production. Further genes quantification analyses revealed that mfAgNPs significantly down-regulated QS-regulated genes, specifically those encoded to the secretion of virulence factors. The results clearly indicated the anti-virulence property of mfAgNPs by inhibiting *P*. *aeruginosa* QS signaling.

Excessive and indiscriminate uses of antibiotics have led to the emergence of multi-drug resistant (MDR) *Pseudomonas aeruginosa*, a notorious opportunistic pathogenic bacterium causing infection in immunocompromised patients^1^,^2^,^3^. The failure of existing antimicrobial agents to control infection makes it crucial to find out alternative approaches to combat resistant *P*. *aeruginosa*^4^. Numerous studies have demonstrated that *P*. *aeruginosa* uses quorum sensing (QS) for forming biofilms and secreting virulence factors^5^. QS signaling relies on the concentration of signal molecules such as *N*-acyl homoserine lactones (AHLs), which bind to their respective transcriptional regulators such as LasR and RhlR^3^. The AHLs are produced intracellularly by LuxI and then exported to the extracellular space. At sufficient concentrations, AHLs bind to their respective receptors (LuxR) which in turn induces transcription of a battery of virulence genes, including biofilm formation, production of toxins, enzymes and other factors that promote pathogenesis^6^.

Anti-QS approach has already shown promise in the battle against pathogenic bacteria^1^,^5^,^7^,^8^,^9^,^10^,^11^. However, developing therapies that stop QS are not straightforward. Some of the most effective chemicals such as halogenated furanones obtained from the Australian macroalgae, *Delisea pulchra* for this purpose are too toxic for human use^12^. However, recently researchers have been utilizing nanotechnology for the development of advanced nanomaterials targeting QS-regulated virulence factors^13^,^14^,^15^. These provide starting points for the development of alternative antibacterial therapies.

Once again the use of silver for treating bacterial diseases has gained importance. However, the use of ionic silver has one major drawback; as they are easily inactivated by complexation and precipitation, thereby limiting the uses of ionic silver as potential antimicrobial^16^. In this respect, noble and functional metal nanoparticles (NPs) are gaining constant research interest due to their potential applications in future bio-nanotechnology. In particular, silver nanoparticles (AgNPs) reveal strong surface plasmon resonant absorption in the ultraviolet and UV-visible region of the electromagnetic spectrum, which has created several interest in the fields of biomedical engineering^17^,^18^. The development of bio-AgNPs, is an emerging area of nanotechnology for their potential application in management of microbial infections^19^,^20^. Recently, nanomaterials alone as well as QS inhibitors-loaded NPs were found to inhibit the production of virulence factors and biofilm formation in *P*. *aeruginosa*, giving clues to check for our synthesized nanomaterial as an anti-virulence agent^21^,^22^.

The culturable microorganisms including fungi and bacteria are now preferred for synthesizing ecofriendly green nano-factories^23^. The cell mass and the secreted metabolites of fungi have been utilized for the reduction of Ag^+^ ions to AgNPs through the oxidoreductases and a shuttle quinone extracellular^23^,^24^. Indeed, the filamentous fungi possess some distinctive advantages over bacteria, including high metal tolerance, wall-binding capacity, intracellular metal uptake capability, ease of handling, and mass cultivation^25^. Testing green nanomaterials is already known for their pharmacological activities, but in this study, we have synthesized the surface protective modified AgNPs using biomass aqueous extract (BAE) of a fungus *Rhizopus arrhizus* BRS-07 (denoted as “mfAgNPs”) inhibiting *P*. *aeruginosa* PAO1 QS and biofilm formation. However, to the best of our knowledge, this is the first report demonstrating anti-QS potential and anti-biofilm activity for mfAgNPs. The results in the attenuation of *P*. *aeruginosa* PAO1 virulence and biofilm by mfAgNPs are reported herein.

## Results

### Characterization of mfAgNPs

**Figure 1:**
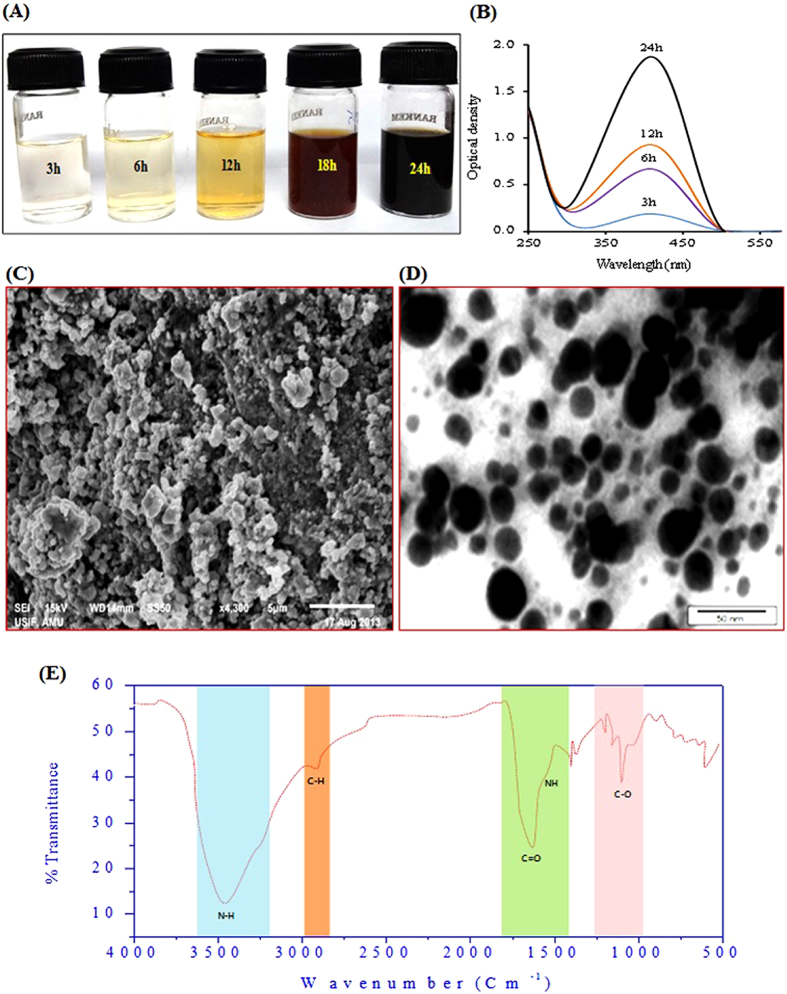
Mycofabrication and characterization of mfAgNPs. (**A**) Biomass aqueous extract of BRS-07 was challenged with 1 mM AgNO_3_ solution upto 24 h and development of color due to the excitation of surface plasmon resonance (SPR) at different time TEM intervals. (**B**) UV-visible absorption spectrum of mfAgNPs at various time intervals. (**C**) SEM micrograph showing the structure of synthesized mfAgNPs. (**D**) TEM analysis showing morphology of mfAgNPs that are polydispersed with a roughly spherical shape, crystalline nature, and agglomeration. The micrograph presenting mfAgNPs of various sizes ranges 5–30 nm. (**E**) FTIR spectrum showing possible interaction between AgNPs and biomolecules of BAE of BRS-07.

It is reported that the active substances of BAE of fungi include oxidoreductases and quinone extracellular process^26^,^27^. These metabolites play a role in the reduction of metal ions and efficient stabilization of nanoparticles. *R*. *arrhizus* BRS-07 was isolated from the roots of Ashawgandha (*Withania somnifera* L.) and characterized at morphological as well as molecular levels (Supplementary figures 1A, B, C, and D). AgNO_3_, upon incubation with BAE of the fungus for 24 h, turned dark brown color (Supplementary figure 2A). The development of color is due to the excitation of surface plasmon resonance (SPR) exhibited by the NPs^28^. Interestingly, no color development was observed, when culture media was incubated with AgNO_3_ for 24 h (Supplementary figure 2B). The intensity of colour was increased with the increase in time of incubation (Fig. 1A). The UV-vis spectrum showed a signature peak of AgNPs at 410 nm due to SPR in AgNPs (Fig. 1B)^17^,^29^. It can be hypothesized that the synthesis of mfAgNPs might have happened due to the reduction of metal ions by metabolites present in BAE of BRS-07.

Supplementary figure 3A shows that the X-ray diffraction (XRD) patterns of mfAgNPs, synthesized using BAE of BRS-07. Obtained data revealed a number of Bragg reflections with 2*θ* values of 38. 4°, 44.5°, 64.6°, and 76.9° sets of lattice planes which may be indexed to the (111) (200), (220), and (311) facets of silver respectively (JCPDS files No. 03-0921). The results thus clearly illustrated that the AgNPs formed were crystalline in nature. The average crystallite size (*d*) of mfAgNPs was calculated by Debye-Scherrer formula: D = *kλ/βCOSθ*. Where *k* = 0.9 is the shape factor, λ is the X-ray wavelength of Cu Kα radiation (1.54 Å), θ is the Bragg’s diffraction angle, and β is the full width at half maximum (FWHM) of plane diffraction peak (111). The average particle size was ~28 nm. The scanning electron microscopy (SEM) analysis was used to examine the structure of mfAgNPs. The SEM micrograph indicated that the surface deposited AgNPs (Fig. 1C). The transmission electron microscopy (TEM) provided further insight into the morphology and size details of the mfAgNPs (Fig. 1D). The NPs synthesized were poly-dispersed agglomerated with a roughly spherical shape and crystalline nature. The analysis of data showed the average size of NPs in range 5–30 nm. The elemental analysis data obtained from energy dispersive X-ray analysis (EDAX) are shown in Supplementary figures 3B and C. A strong peak in silver region confirmed the formation of AgNPs. Metallic silver nanomaterials generally exhibit typical optical absorption peak approximately at 3KeV due to SPR. Moreover, the occurrence of carbon and oxygen peaks also suggested the presence of organic moieties on the surface of NPs which might have come from the biomass of BRS-07.

Fourier transform infrared spectroscopy (FTIR) measurement was carried out to identify the possible biomolecules in BAE of BRS-07 involved in the capping and stabilization of mfAgNPs. Fig. 1E shows the FTIR spectrum of mfAgNPs. The major absorbance peaks present in the spectrum of mfAgNPs showed signature absorbance peaks at 3433.22 cm^−1^, 2940.55 cm^−1^, 1639.97 cm^−1^, 1381.12 cm^−1^, and 1085.32 cm^−1^, respectively. The amide associations between amino acid residues in proteins gave rise to the well-known marks in the infrared region of the electromagnetic spectrum. The peaks seen at 3433.22 cm^−1^ and 2940.55 cm^−1^ were referred to the stretching vibrations of primary and secondary amines, respectively assigned; although their corresponding bending vibrations were observed at 1639.97 cm^−1^ and 1381.12 cm^−1^, respectively. The peak at 1085.32 cm^−1^ can be assigned to the C-N stretching vibration of aliphatic amines. Earlier FTIR study in one of the investigation has reported that the carbonyl groups, amino acid residues and peptides have strong affinity to silver^26^. Moreover, free amine groups or cysteine residues in proteins bind to nanoparticles^30^, thereby possibly capping and stabilizing the AgNPs.

### Assessment of Ag^+^ ions release from mfAgNPs

Confirmation of slow release of Ag^+^ ions released from mfAgNPs into culture media was obtained as the results indicated about 39% release of total Ag^+^ in 5 days (Supplementary figure 4). Thus, it pointed out the role of deposited proteins on the surface of NPs in improving the sustainable release of Ag^+^ ions.

### Localization of mfAgNPs

**Figure 2:**
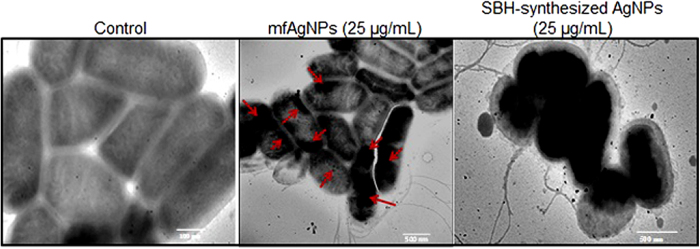
Examination of mfAgNPs internalization into PAO1 cells by TEM. Red arrows indicate the accumulation of mfAgNPs inside the cells.

To examine the interaction between mfAgNPs with PAO1 cells, next we examined whether our synthesized NPs internalized into the cells of PAO1. For this, cells were treated with 25 μg/mL of mfAgNPs or distilled water (DW), as vehicle for 12 h, and then analyzed by TEM. A clear localization of the NPs into the cells was observed and PAO1 cells noticed as normal (Fig. 2). However, abnormal cells were observed, when exposed to 25 μg/mL of SBH-synthesized AgNPs which clearly indicate the toxicity of NPs.

### Anti-QS activity

**Figure 3:**
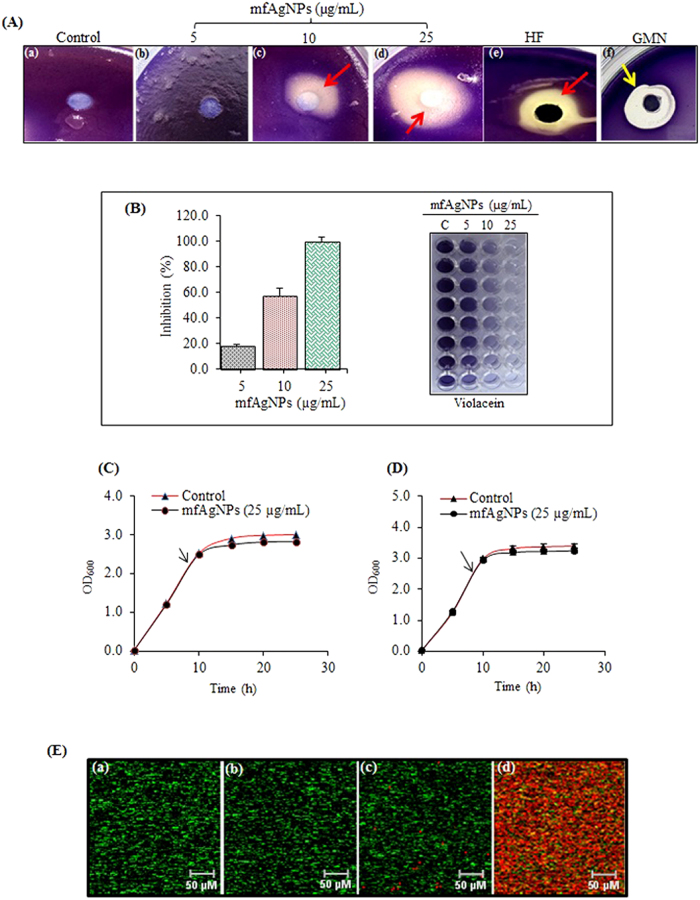
Assessment of anti-QS activity of mfAgNPs. (**A**) A bioindicator strain *C. violaceum* 12472 was used for examining anti-QS activity. Treatments include (a) solvent vehicle (H_2_O), (b-d) various mfAgNPs concentrations including 5, 10, and 25 μg/mL. (e) 10 μg/mL of HF. (f) 10 μg/mL of GNM. Inhibition of violacein pigment production (e.g. a colorless and opaque halo zone) indicates anti-QS effect (red arrows), while a clear and transparent zone shows cidal effect (yellow arrows). (**B**) Inhibition of violacein production at different mfAgNPs concentrations (0, 5, 10, and 25 μg/mL) was quantified spectrophotometrically with OD at A585 nm. Growth curves of (**C**) CV026 and (**D**) PAO1 at different concentrations of mfAgNPs for 25 h. Error bars indicate the SD of 6 measurements. (**E**) Examination the effect of mfAgNPs on PAO1 cell viability using CLSM. For this, cells were grown in the presence of (a) untreated control, (b) solvent vehicle (H_2_O), (c and d) 25 and 50 μg/mL of mfAgNPs for 24 h and stained with SYTO-9 green for live cells and PI red for dead cells.

The possibility of QS attenuation by mfAgNPs was firstly investigated by disc diffusion anti-QS assay using a bio-indicator strain *Chromobacterium violaceum* 12472, which produces the AHL-regulated violet-colored ‘violacein’ pigment^5^,^31^. In this assay, the development of violacein represents AHL-dependent QS signaling, while the inhibition of violacein indicates the anti-QS activity via attenuation of AHL production. A concentration dependent inhibitory effect of the mfAgNPs on violacein production was observed. The highest inhibition was recorded at 25 μg/mL, while no activity was examined with 5 μg/mL (Fig. 3A-c,d). Control discs containing halogenated furanone (HF; C-30) and gentamycin (GMN) were included. As expected, a zone of growth inhibition was detected with GMN (Fig. 3A-e), while an opaque zone of QS inhibition was seen with the HF, and no inhibition was apparent with DW (Fig. 3A-f). However, 50 μg/mL of mfAgNPs and 25 μg/mL of SBH-synthesized AgNPs showed the growth inhibitory effect against *C. violaceum* (Supplementary figure 5), suggesting the surface modification of mfAgNPs by proteins of BRS-07. Similar results were observed in colorimetric measurement of violacein production as 100% inhibition was observed by 25 μg/mL of mfAgNPs (Fig. 3B). Next, we sought to examine the effect of mfAgNPs (25 μg/mL) on growth of *C*. *violaceum* CV26 and PAO1. The obtained results revealed that the cell densities of bacteria did not significantly differ between untreated control and cultures exposed to 25 μg/mL of mfAgNPs (Fig. 3C,D). In addition, MTT (3-(4,5-dimethylthiazol-2-yl)-2,5 diphenyltetrazolium bromide) toxicity assay was also employed against PAO1 and human epithelial cells which showed non-toxic effect of mfAgNPs (Supplementary figures 5C and D), as evident from the confocal laser scanning microscopy (CLSM) analysis of PAO1 (Fig. 3Ea–d). However, a strong toxicity effect was observed, when cells exposed to 50 μg/mL of mfAgNPs (Fig. 3E-d). The results obtained demonstrated that mfAgNPs could have interfered QS without any toxic effect via attenuation of AHL production.

### Inhibition of virulence by mfAgNPs

Virulence factors namely LasA protease, LasB elastase, siderophores, rhamanolipid, and alginate were analyzed to evaluate the effects of mfAgNPs on PAO1 virulence. Production of these virulence factors was inhibited by mfAgNPs in a concentration dependent manner (0–25 μg/mL): 15–86% inhibition for LasA protease, 22–86% inhibition for LasB elastase, 18–96% suppression for pyocyanin, 14–95% suppression for pyoverdin, 10–92% suppression for pyochelin, and 10–70% inhibition for rhamnolipid (Supplementary figures 7A–F). In addition, inhibition of rhamnolipid production in culture filtrate and cell bounded by mfAgNPs was also observed using thin-layer chromatography (TLC) and agar plate assay (Supplementary figures 7G and H). Rhamnolipids have been reported to play important role in QS-mediated swarming motility and biofilm dispersion from the infection site, which is facilitated by reduced friction through the action of rhamnolipid detergent^32^. Obtained results from agar plate methods revealed that the mfAgNPs inhibited swimming and swarming behaviors of PAO1 (Supplementary figures 8A and B). Alginate, an exopolysaccharide constitutes the major portion in *P. aeruginosa* biofilm matrix and its production is known to be controlled by QS. The effect of mfAgNPs was examined for their potential to diminish the production of alginate. The production of alginate was reduced significantly as the concentration of mfAgNPs increased. Reduced alginate production by 67% was observed at 25 μg/mL of mfAgNPs (Supplementary figure 7I).

#### Reduction of QS-regulated virulence gene expression by mfAgNPs

**Figure 4:**
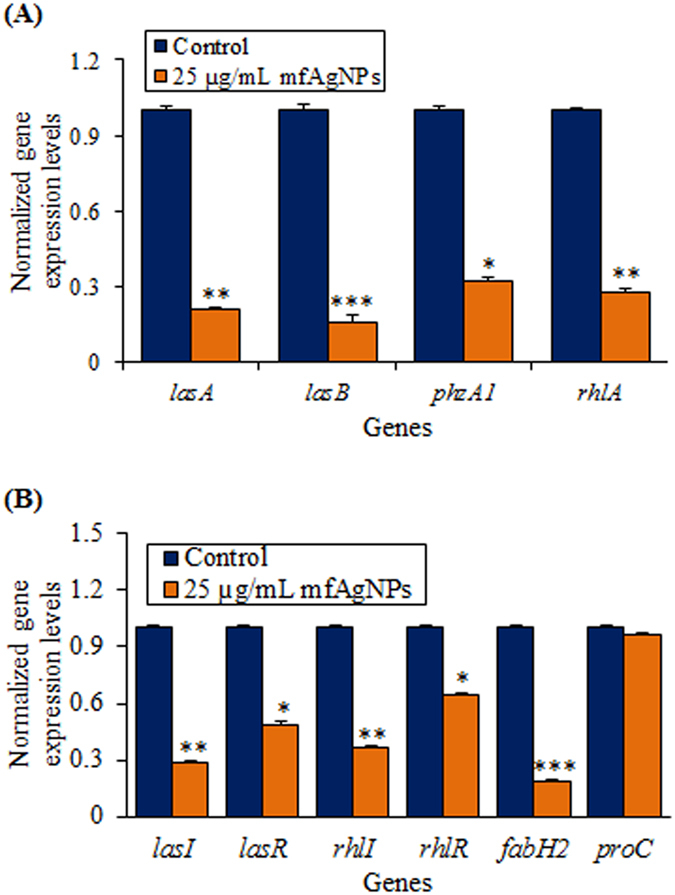
Effect of mfAgNPs on the expression of QS-controlled genes of PAO1. (**A**) The expression of genes namely *lasA*, *lasB*, *phzA1*, and *rhlA*, *lasI*, *lasR*, *rhlI*, *rhlR*, *fabH2*, and *proC* (housekeeping gene) was assessed in mfAgNPs treated or untreated control cDNA by RT-qPCR. The relative magnitude of gene expression level was defined as the copy number of cDNA of each gene in the planktonic cells normalized by the copy number of cDNA of the corresponding gene in planktonic cells without mfAgNPs. Error bars indicate the SD of 3 measurements. ***P < 0.001 Vs control. **P < 0.01 Vs the control. *P < 0.05 Vs control. The same volume of H_2_O was added to the control treatments.

To further explore anti-QS potential of mfAgNPs, expression of QS-regulated genes encoding virulence factors of P. aeruginosa PAO1 was studied. The expression of genes such as *lasA*, *lasB*, *phzA1*, and *rhlA* of PAO1 planktonic cells was analyzed to identify the genes targeted by mfAgNPs and to investigate the molecular mechanism that reduces virulence when mfAgNPs are supplemented. RT-qPCR analysis was to compare the gene expression between PAO1 cells treated and untreated with mfAgNPs. As shown in Fig. 4A, all four genes were significantly down-regulated in the planktonic cells, when treated with mfAgNPs. The expression of *lasA* and *lasB* were repressed about 79 and 84%, respectively by 25 μg/mL of mfAgNPs. Although the expression of *phzA1* and *rhlA* were comparatively less reduced by 68 and 72%, respectively as compared to *lasA* and *lasB*.

The effects of mfAgNPs (25 μg/mL) were also examined on *P*. *aeruginosa* QS regulon LasIR-RhlIR using β-galactosidase reporter assay. Table 1 shows a significant inhibition of LasI transcriptional activity by 71%, while 50% down-regulation of LasR was observed. In addition, mfAgNPs also decreased the levels of RhlI and RhlR by 64 and 55%, respectively. The down expressions of Las and Rhl in transcriptional activity were further confirmed by RT-qPCR. Recorded data confirmed that the down-regulation of genes such as *lasI*, *lasR*, *rhlI*, *rhlR*, and *fabH2* by 71, 51, 63, 36, and 81%, respectively by 25 μg/mL of mfAgNPs (Fig. 4B). The expression of the *proC* housekeeping gene was not strongly affected by mfAgNPs.

### Inhibition of AHLs production by mfAgNPs

**Figure 5:**
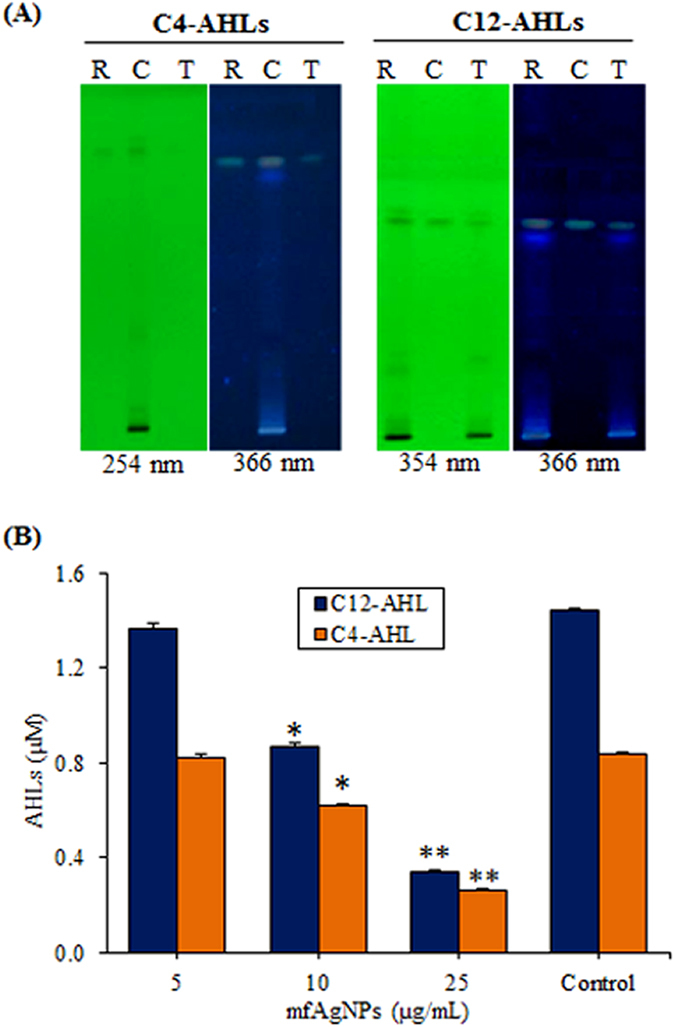
Impact of mfAgNPs on the production of AHLs in PAO1. Quantification of C12-AHL and C4-AHL in mfAgNPs treated or untreated for 48 h by (**A**) HPLTC on silica gel 60 F_254_ TLC plates using a mobile phase of methanol-water-acetic acid (8:1.5:0.5) and by (**B**) ES-MS analysis. R, reference compound. C, untreated control. T, 25 μg/mL of mfAgNPs. Error bars indicate the SD of 3 measurements. **P < 0.01. *P < 0.05.

The *lasI* and *rhlI* genes are required for the synthesis of AHLs and the activation of LasR-RhlR regulon in *P*. *aeruginosa*. We were quite interested to probe if AHLs production would be inhibited in the PAO1 culture fluid after the treatment of mfAgNPs. Thus, PAO1 exposed to mfAgNPs was analyzed for the content of AHLs by HPTLC. Inhibition of C12-AHL and C4-AHL productions were observed, when PAO1 was exposed to 25 μg/mL of mfAgNPs (Fig. 5A). Fig. 5B shows the quantification of AHLs by electrospray mass spectroscopy (ES-MS). Production of AHLs was inhibited by mfAgNPs in a dose dependent manner (0–25 μg/mL): 5–76% reduction for C12-AHL and 2–69% inhibition for C4-AHL when compared to their untreated controls.

### Anti-biofilm activity of mfAgNPs

**Figure 6:**
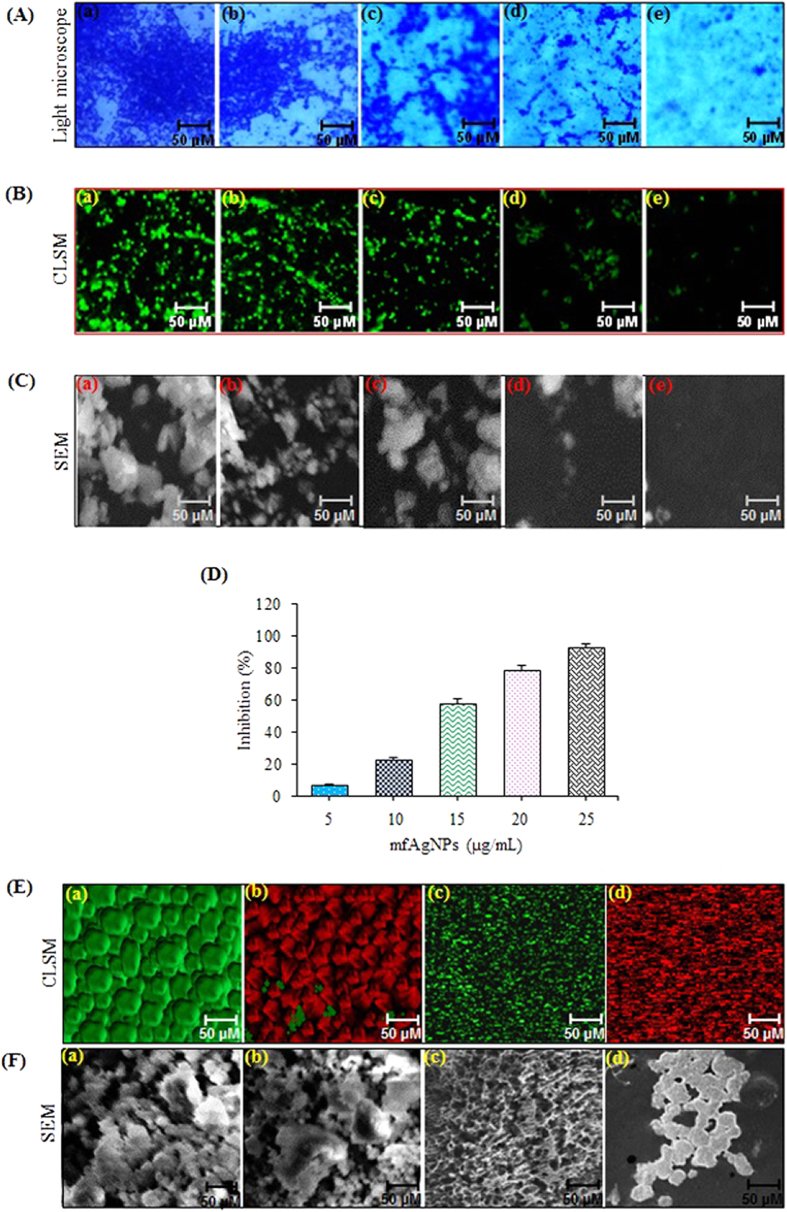
Anti-biofilm activity of mfAgNPs and their effect along with tobramycin on PAO1 biofilms. Images of (**A**) CV-staining light microscope, (**B**) SYTO-9 staining CLSM, and (**C**) uranyl acetate-lead citrate staining SEM biofilms, treated with different mfAgNPs concentrations: (a) 0 μg/mL, (b) 5 μg/mL, (c) 10 μg/mL, (d) 15 μg/mL, and (e) 25 μg/mL for 48 h. (**D**) Biofilm formation at indicated concentrations of mfAgNPs for 48 h in microtiter plate and detail methodology is mentioned in Supplementary methods. Error bars indicate the SD of 3 measurements. (**E**,**F**) Effect of mfAgNPs on tolerance of biofilms to tobramycin was assessed by CLSM and SEM. Images of biofilm formed in flow chambers for 72 h and then treated with indicated concentrations of mfAgNPs for next 24 h and determined the cell viability using Bacterial Cell Viability Kit. (**F**) Same cells were also processed for SEM analysis and photographed. Treatments include (a) H_2_O, (b) 350 μg/mL of tobramycin, (c) 25 μg/mL of mfAgNPs, and (d) 350 μg/mL of tobramycin plus 25 μg/mL of mfAgNPs.

Microscopic analysis was used to provide initial clue of anti-biofilm property of mfAgNPs, therefore we performed crystal violate (CV) staining assay. A dark CV stained biofilm was observed in untreated control, while a dose-dependent visible reduction in number of microcolonies was examined in mfAgNPs-treated PAO1 biofilm (Fig. 6A-a–e). Additionally, mfAgNPs also disrupted the architecture of biofilm too, as it was more evident from CLSM (Fig. 6B-a–e) and SEM (Fig. 6C-a–e) micrographs. The effect of mfAgNPs on PAO1 biofilm formation was also evaluated using a static biofilm assay. Biofilm formation was inhibited 7–93% by mfAgNPs in a dose dependent manner as shown in Fig. 6D. HF, a positive control was also found to inhibit biofilm formation by 11–96% at the concentrations of 5–25 μg/mL (Supplementary figure 9).

The efficacy to eradicate SDS-resistant biofilms of PAO1 by mfAgNPs alone or in combination with tobramycin was also evaluated. Tobramycin, an aminoglycoside antibiotic derived from *Streptomyces tenebrarius* is routinely used to treat various types of bacterial infections^33^. The mfAgNPs were found to be very effective in eradicating the sodium dodecyl sulfate (SDS)-resistant biofilms, in contrast to the untreated control (Supplementary figure 10). It was further confirmed that the ability of *P*. *aeruginosa* to form the typical mushroom like structured and SDS-resistant biofilms is regulated by QS. Biofilms were also allowed to form for 72 h in flow chambers and subsequently these biofilms were treated for the next 24 h with 25 μg/mL of mfAgNPs alone and mfAgNPs plus 250 μg/mL of tobramycin. Figure 6E-a–d shows CLSM images of PAO1biofilms formed in flow chamber. The biofilm treated with mfAgNPs plus tobramycin was observed to be more susceptible for cell death than tobramycin alone as analyzed by Bacterial Cell Viability Kit (Fig. 6E-d). In presence of mfAgNPs, tobramycin efficiently penetrated and killed 100% cells of the biofilm. However, the biofilm treated with tobramycin, only cells at the surface of the biofilm were killed (Fig. 6E-b). These biofilms were also analyzed by SEM analysis which showed similar effects (Fig. 6F-a–d). Our results are consistent with the previous report, wherein HF acted synergistically with tobramycin to eradicate *P*. *aeruginosa* biofilms^34^.

## Discussion

**Figure 7:**
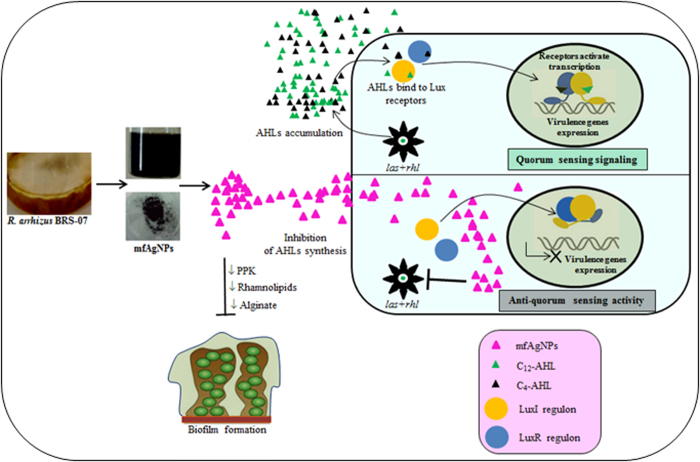
Possible anti-QS mechanism of mfAgNPs. *P*. *aeruginosa* uses Las-Rhl systems to produce signaling molecules (i.e. AHLs) intracellularly which then exported to the extracellular space to assess their cell density. Once a pre-determined level is exceeded, LuxR-AHLs complex activates the expression of target genes and this mechanism is called as QS. After cellular internalization of mfAgNPs might likely interact with PAO1QS systems, thereby inhibiting the LasIR-RhlIR-mediated AHLs production. In the absence of AHLs, LuxI and LuxR receptors do not bind to DNA, thereby inhibiting the expression of targeted genes which encode virulence factors and biofilms.

The present study clearly demonstrated that mfAgNPs attenuated *P*. *aeruginosa* QS systems and biofilm formation without a significant effect on its growth (Fig. 7; Supplementary figures 6A, B and D). Production of violacein pigment by *C*. *violaceum* 12472 is regulated by AHL-mediated QS system. Inhibition of violacein production by mfAgNPs clearly confirmed their anti-QS activity in a concentration dependent manner. This is in accordance with the previous studies on the inhibition of violacein production by anti-QS nanomaterials^35^,^36^. In addition, mfAgNPs inhibited the production of extracellular virulence factors including proteases, siderophores, rhamnolipid and alginate of PAO1. Moreover, higher stability of mfAgNPs was noticed at room temperature upto 330 days of incubation as well as in culture media for 72 h as compared to SBH-synthesized AgNPs (Supplementary figures 11A–E).

Anti-QS and anti-biofilm activities of mfAgNPs were initiated with down-regulation of LasIR-RhlIR transcriptional activity by mfAgNPs, which was evident by the β-galactosidase assay. Recently, our group reported the anti-QS potential of *Lagerstroemia speciosa* fruit extract through inhibition of transcriptional activities of LasIR and RhlIR in *P*. *aeruginosa* PAO1^5^. LasIR and RhlIR are two principle QS systems that have been characterized in *P*. *aeruginosa* which is reported to cause severe infections of the respiratory tract. LasI and RhlI synthases are required for the production of C12-AHL and C4-AHL, respectively. The C12-AHL controls the production of tissues destructive virulence factors including elastase, exotoxin, protease, and alkaline phosphatase and also activates the second QS system. C4-AHL on the other hand takes part in controlling the expression of several genes encoding elastase, alkaline phosphatase, and pyocyanine. In the present investigation, reduced production of AHLs by mfAgNPs might have interfered with the normal secretion of PAO1 virulence factors such as proteases and siderophores. A similar anti-QS effect has been examined with a HF^37^. The probability of mfAgNPs to inhibit the production of AHLs was also supported by the *C*. *violaceum* 12472-based anti-QS colorimetric assay in which the AHL-mediated production of violacein pigment was inhibited by increasing mfAgNPs concentration. Likewise, inhibition in AHLs production by anti-QS extract of medicinal plants was reported by Adonizio and colleagues in PAO1^1^.

Anti-QS activity of mfAgNPs also appears to inhibit with normal QS circuitry in PAO1 via the molecular mechanism. At a high cell density, the AHL-LasR complex acts as transcriptional activator and triggers the expression of targeted genes within QS regulon such as *lasA*, *lasB*, *lasI*, *lasR*, *rhlI*, *rhlR*, *rhlA*, *phzA1*, and *fabH2*. The down expression of these genes in the Rt-qPCR results supports our speculation for the anti-virulence activity of mfAgNPs. Reduction of these genes might interfere with normal production of several virulence factors such as LasA protease, LasB elastase, pyocyanin, and rhamnolipid via the actions of the *lasAB*, *phzA1*, *rhlA* operons, respectively. The expression of *rhlR* triggers the production of the AHL signal receptor, RhlR. The C4-AHL and RhlR is the transcriptional activator for the rhlAB and fabH2 operons. The fabH2 operon has been reported to enhance pathogenicity of *P. aeruginosa* by regulating AHLs production^38^. Therefore, down-regulation of *rhlR* by mfAgNPs might cause a decrease in the RhlR production that can result in a decrease in the rhlAB operon and *rhlI* production. Moreover, reduced expression of *fabH2* gene also recorded with treatment of mfAgNPs. Our results are consistent with the report of Hentzer and Givskov^34^, wherein HFs have shown anti-QS activity in PAO1 by inhibiting the expression of *fabH2* gene.

The first evidence that QS regulates biofilm formation, a key player in the pathogenesis of several bacteria was reported in *P*. *aeruginosa*^39^. Therefore, targeting deactivation of QS could be a good preventive strategy for biofilm formation in PAO1. Our results indicated that the mfAgNPs not only inhibited biofilm formation in PAO1, but also reduced the microcolonies formation, and altered biofilm morphology too. As reported by the previous studies describing the effect of QS inhibitors, our mfAgNPs also inhibited biofilm formation in PAO1^4^,^8^,^15^. Swimming motility was also decreased with increasing concentrations of mfAgNPs. Flagella-mediated swimming motility is known to confer enhanced biofilm formation by instigating the cell-to-surface attachment^40^. Therefore, inhibition of swimming motility could be one of the reason for decreased biofilm formation. Musthafa and colleagues also examined a similar effect wherein phenylacetic acid inhibited the biofilm formation of PAO1 by interfering with its swimming motility^41^.

It is well documented that increased sensitivity towards antibiotics depends on the process of QS. Reducing thickness and cell viability of the matured biofilms by mfAgNPs eventually led to increased susceptibility of PAO1 biofilms to tobramycin. It could also be well associated with the inhibition of rhamnolipid and alginate production, because they are considered as important factors for maintaining biofilm architecture and maturation resistance to antibacterials. Rasmussen *et al.* and Singh *et al.* have also been revealed the possible role of anti-QS compounds and plant extracts to increase sensitivity of matured biofilms of PAO1 to tobramycin^5^,^38^. The results suggests the dual action of mfAgNPs in inhibiting the biofilm formation at the initiation level and enhancement in the susceptibility of PAO1 biofilms to conventional antibiotic might be due to the inhibition of LasR by mfAgNPs^11^.

The present study reports that mfAgNPs could attenuate virulence and biofilm formation of *P*. *aeruginosa* PAO1 which is an essential criteria for enhancing the antibiotic efficiency and pathogenicity of bacterial pathogens. Anti-virulence property of mfAgNPs might be due to inhibition of LasR-RhlR. Thus, the mfAgNPs present a possibility for use as potential nanomaterials to combat *P. aeruginosa* infections by interrupting QS in healthcare environments in the future.

## Methods

### Mycofabrication of AgNPs

At first, BRS-07 was cultured in potato dextrose broth (PDB) having pH 6.5 at 27 ± 1 °C under constant shaking (150 rpm). After incubation of 72 h, biomass was collected by centrifugation at 5000 rpm for 10 min and washed thoroughly with sterilized DW to facilitate removal of any adhered materials/or media components. The collected wet biomass (15 g) was transferred to DW (100 mL) and incubated for 24 h at 4 °C. The solution was centrifuged at 5000 rpm for 10 min and obtained BAE was collected. For biosynthesis of NPs, BAE was challenged with 1 mM of AgNO_3_ and incubated at 30 ± 0.5 °C under constant shaking at 100 rpm for 24 h. The color change in reaction mixture was routinely monitored both by visual inspection and absorbance measurements using UV-vis spectrophotometer (Perkin Elmer Life and Analytical Sciences, CT, USA). The synthesized nanomaterial was denoted as “mfAgNPs”.

### Characterization of mfAgNPs

The SPR spectra of synthesized AgNPs in reaction mixture were recorded at various time intervals using UV-vis spectrophotometer in the wavelength ranged from A_200_ to A_800_ nm. The mfAgNPs were freeze dried and used for further characterization. The crystalline nature of mfAgNPs, powered sample was analyzed by MiniFlex™ II benchtop XRD (Rigaku) with Cu-Kα Radiation (λ = 1.54178 Å) in the range of 20–80° at 40 kV^42^. The surface morphological property of mfAgNPs was analyzed by SEM (JSM6510LV, JEOL Japan) and transmission electron microscopy (JEM2100, JEOL, Japan) using powder and aqueous samples, respectively. For elemental analysis, Oxford Instruments INCAx-sight EDAX spectrometer equipped with SEM was used^43^. The functional nature and surface modification of mfAgNPs were ascertained by FTIR spectrum two (Perkin Elmer Life and Analytical Sciences, CT, USA) at room temperature in the range of 400–4000 wavenumber (cm^−1^). For this, powdered sample was mixed with spectroscopic grade potassium bromide (KBr) in the ratio of 1:100 in diffuse reflectance mode at a resolution of 4 cm^−1^ in KBr pellets^44^. The functional and thermal stability of mfAgNPs was examined by Pyris1 thermogravimetric analyzer (TGA; Perkin Elmer Life and Analytical Sciences, CT, USA) in a flowing air atmosphere at the temperature ranged of 50–700 °C with a heating rate of 10 °C/min^45^.

### Determination of anti-QS activity

#### Bacterial strains and culture conditions

Anti-QS activity of the mfAgNPs was determined using a bio-indicator strain of *C. violaceum* ATCC 12472. This bacterium produces a purple colored pigment ‘violacein’ which is controlled by QS systems^37^,^46^. However, *C*. *violaceium* ATCC 026 (CV026) is a mini-*Tn5* mutant of *C*. *violaceum* 12472, that is unable to synthesize its own AHLs but it retains the ability to respond to exogenous AHLs^37^. CV026 is used for growth curve experiment. *P*. *aeruginosa* PAO1 was generously provided by Prof. Kalai Mathee, Florida International University, USA. *C*. *violaceum* was grown in Luria-Bertani broth (LB) medium, while nutrient broth (NB) was used for PAO1 cultivation. The medium was solidified with 1.5% agar when required and supplemented with appropriate antibiotics, such as kanamycin (20 μg/mL) for *C. violaceum* 12472 and CV026, and potassium tellurite (150 mg/L) for *P*. *aeruginosa* PAO1.

#### Intracellular NPs accumulation assay

Cells were collected from overnight grown culture of PAO1, washed twice with 0.8% NaCl, and resuspended in NB media at 2 × 10^8^ cells/mL. The mfAgNPs were added to the culture and incubated at 30 °C with constant shaking (200 rpm) for 12 h. Cells were collected by centrifugation, washed twice, and fixed in the fixative solution (sodium cacodylate buffer, pH 7.2, containing 4% polyoxymethylene) at 4 °C for 24 h. The samples for TEM analysis were prepared by placing a drop of PAO1cell suspension in DW onto amorphous carbon-coated copper grids, and DW was allowed to evaporate slowly at room temperature. The samples coated grids were analyzed at 500 nm scale by TEM (JEM2100, JEOL, Japan) with an accelerating voltage of 80 kV.

#### Anti-QS activity assay

A standard disc diffusion assay was used to determine the anti-QS activity of mfAgNPs^47^. *C. violaceum* 12472 was grown overnight in LB broth medium containing the appropriate antibiotic. Hundred microliter of the inoculum was mixed with 5 mL of molten LB agar (0.3%) and immediately poured over the surface of pre-poured LB agar plate. After solidification, sterilized paper discs were placed on the surface of medium. Various concentrations of the mfAgNPs were applied on the discs and incubated overnight at 30 ± 1 °C. A colorless, opaque, and halo zone (i.e. inhibition of violacein production) around the disc was considered as anti-QS activity of test sample.

#### Violacein production assay

Quantification of violacein pigment production in *C*. *violaceum* 12472 was carried out as described earlier^1^,^47^. Briefly, mfAgNPs-treated and untreated cultures were lysed by adding 10% of SDS detergent. Violacein content was fractionated with water saturated n-butanol and recorded the absorbance at A_585_ nm.

#### Growth kinetics assay

Overnight grown cultures of *C*. *violaceum* CV026 and *P*. *aeruginosa* PAO1 were diluted 100-folds into minimal medium. The absorbance at A_600_ nm was monitored at every 45 min, until an absorbance at A_600_ nm of 1.6 was reached. Bacterial cultures were then divided into 28 mL of aliquots, to which 2 mL of minimal medium (control) or 2 mL of mfAgNPs (25 μg/mL; dissolved in a medium) were added. The absorbance at A_600_ nm was monitored at 5 h of intervals until a final time point of 25 h. All absorbance values were verified at 1/10 dilution for greater accuracy^46^.

#### Cell viability assay

Glass cover slips were placed into 6-well plate containing culture medium and PAO1 (1 × 10^7^ cells/mL). After incubation of 24 h at 30 °C, cells were stained with SYTO-9 and propidium iodide (PI) according to the manufacturer’s protocol (Molecular Probes, Invitrogen). Then, cells were analyzed by CLSM (LSM510, Germany). The ratios between the green and the red fluorescence were compared to assess the cytotoxicity of mfAgNPs.

#### Quantitation of AHL production

A method of Shaw *et al.* was adopted for the extraction and isolation of AHLs^48^. Briefly, PAO1 was cultured in 2.5 L of AB minimal medium at 30 °C under constant shaking. After incubation of 18 h, culture broth was centrifuged at 6000 g for 10 min and obtained supernatant was extracted with acidified ethyl acetate (7 : 3). The solvent was evaporated under reduced pressure using a rotary evaporator (BUCHI, USA) at 40 °C. The dried powder was reconstituted in acetonitrile and quantified by ES-MS. Peak intensities for C4-AHL (m/z = 172) and C12-AHL (m/z = 148 and 298) and their sodium adducts (m/z = 194 and 230, respectively) were combined and converted to concentrate using a standard curve generated from the pure compounds^1^.

#### β-galactosidase activity assay

Transcriptional activity of QS-related gene promoters was assayed using PAO1-derived strains harboring promoter *lac*Zfusions: PlasI-_lacZ_(pPCS223), PlasR-_lacZ_(pPCS1001), PrhlI-_lacZ_(pLPR1), and PrhlR-_lacZ_(pPCS1002). A promoter-less lacZ fusion strain (pLP170) was used as a control. Cells were grown in AB minimal media and monitored under the same conditions as PAO1 grown. The mfAgNPs were added once the culture growth reached to an OD_600_ of 1.7^1^.

*RT-qPCR assay.* The solvent vehicle (DW) and mfAgNPs-treated PAO1 cells were frozen in liquid nitrogen and disrupted. Total RNA was extracted using TRIZOL reagent (Sigma, USA). DNA contamination from the sample was eliminated by giving DNase treatment for 1 h (Amplification Grade DNase I from Sigma). cDNA was synthesized from 250 ng of total RNA using the Reverse Transcription System (Promega) and random hexamers according to the Promega’s instructions. Primers were designed as reported by Spangenberg^49^. RT-qPCR was done using Eppendorf Real Plex System with two step PCR program: 95 °C for 10 min (denaturation at 95 °C for 15 s and annealing at 60 °C for 1 min) and 40 cycles. The n-fold change in mRNAs expression was determined according to the method of 2^−∆∆ct^ with housekeeping gene *proC*, used as the internal control.

#### Assessment of biofilm formation

Biofilms of PAO1 were cultivated on sterilized glass-cover slides in sterilized AB minimal medium^37^. The mfAgNPs (25 μg/mL) were added to AB minimal medium and the development of biofilms for 48 h was studied by CV staining, SEM, and CLSM. Tolerance of biofilms to tobramycin in presence of mfAgNPs was assessed by introducing tobramycin (350 μg/mL) to the influent medium to 3 days old PAO1 biofilms^5^. After incubation of 24 h with mfAgNPs (25 μg/mL), the biofilms were examined by CLSM, Bacterial cell viability in biofilm cultures was also assessed using the Live/Dead BacLight Bacterial Viability Staining Kit (Invitrogen, UK). The stain stock solutions of SYTO 9 (20 mM) and PI (20 mM) were diluted 2000-fold in AB minimal medium and injected into the flow channels. The staining was allowed to progress for 15 min with the medium flow arrested and cells were analyzed by CLSM^38^. These biofilms were also analyzed by SEM. The detail protocols of CV staining and SEM were presented in supplementary methods section.

### Statistical analysis

All the statistical analysis was performed using student *t*-test and *p* < 0.05 was considered significant.

## Additional Information

**How to cite this article**: Singh, B. R. *et al.* Mycofabricated biosilver nanoparticles interrupt *Pseudomonas aeruginosa* quorum sensing systems. *Sci. Rep.* **5**, 13719; doi: 10.1038/srep13719 (2015).

## Supplementary Material

Supplementary Information

## Figures and Tables

**Table 1:** Effect of mfAgNPs on transcriptional activity of lasIR-rhlIR.

Concentration (μg/mL)	Transcriptional activity^a^			
	lasI	lasR	rhlI	rhlR
5	3125 ± 62.6	4733 ± 84.3	4357 ± 101.3	6788 ± 175.2
10	2537 ± 48.4^x^	3927 ± 89.3^x^	3155 ± 99.2^x^	5584 ± 201.1^x^
25	949 ± 30.6^y^	2416 ± 54.7^y^	1581 ± 51.7^y^	3751 ± 169.3^y^
Control	3271 ± 39.5	4855 ± 45.2	4518 ± 62.6	6893 ± 129.4

^a^Transcriptional activity was measured via β-galactosidase activity of the lacZ gene fusion products and expressed as Miller Units.

^x^Indicates significance at *p* < 0.05.

^y^Indicates significance at *p* < 0.10.

## References

[b1] Adonizio A., Kong K. F. & Mathee K. Inhibition of quorum sensing-controlled virulence factor production in Pseudomonas aeruginosa by South Florida plant extracts. Antimicrob Agents Chemother 52, 198–203 (2008). 17938186 10.1128/AAC.00612-07 PMC2223872

[b2] Lieberman D. Pseudomonal infections in patients with COPD: epidemiology and management. Am J Respir Med 2, 459–468 (2003). 14719985 10.1007/BF03256673

[b3] Bhuwan M., Lee H. J., Peng H. L. & Chang H. Y. Histidine-containing phosphotransfer protein-B (HptB) regulates swarming motility through partner-switching system in Pseudomonas aeruginosa PAO1 strain. J Biol Chem 287, 1903–1914 (2012). 22128156 10.1074/jbc.M111.256586 PMC3265871

[b4] Ramalingam V. *et al.* Biosynthesis of silver nanoparticles from deep sea bacterium Pseudomonas aeruginosa JQ989348 for antimicrobial, antibiofilm, and cytotoxic activity. J Basic Microbiol (2013). 10.1002/jobm.201300514 24136453

[b5] Singh B. N., Singh H. B., Singh A., Singh B. R., Mishra A. & Nautiyal C. S. Lagerstroemia speciosa fruit extract modulates quorum sensing-controlled virulence factor production and biofilm formation in Pseudomonas aeruginosa. Microbiology 158, 529–538 (2012). 22117007 10.1099/mic.0.052985-0

[b6] Mittal R., Sharma S., Chhibber S. & Harjai K. Contribution of quorum-sensing systems to virulence of Pseudomonas aeruginosa in an experimental pyelonephritis model. J Microbiol Immunol Infect 39, 302–309 (2006). 16926976

[b7] Arevalo-Ferro C. *et al.* Identification of quorum-sensing regulated proteins in the opportunistic pathogen Pseudomonas aeruginosa by proteomics. Environ Microbiol 5, 1350–1369 (2003). 14641579 10.1046/j.1462-2920.2003.00532.x

[b8] Hentzer M. *et al.* Inhibition of quorum sensing in Pseudomonas aeruginosa biofilm bacteria by a halogenated furanone compound. Microbiology 148, 87–102 (2002). 11782502 10.1099/00221287-148-1-87

[b9] Muh U., Schuster M., Heim R., Singh A., Olson E. R. & Greenberg E. P. Novel Pseudomonas aeruginosa quorum-sensing inhibitors identified in an ultra-high-throughput screen. Antimicrob Agents Chemother 50, 3674–3679 (2006). 16966394 10.1128/AAC.00665-06 PMC1635174

[b10] Anwar H. & Costerton J. W. Enhanced activity of combination of tobramycin and piperacillin for eradication of sessile biofilm cells of Pseudomonas aeruginosa. Antimicrob Agents Chemother 34, 1666–1671 (1990). 2126686 10.1128/aac.34.9.1666 PMC171902

[b11] Kim H. S., Lee S. H., Byun Y. & Park H. D. 6-Gingerol reduces Pseudomonas aeruginosa biofilm formation and virulence via quorum sensing inhibition. Sci Rep 5, 8656 (2015). 25728862 10.1038/srep08656 PMC4345325

[b12] Gram L., de Nys R., Maximilien R., Givskov M., Steinberg P. & Kjelleberg S. Inhibitory Effects of Secondary Metabolites from the Red Alga Delisea pulchra on Swarming Motility of Proteus mirabilis. Appl Environ Microbiol 62, 4284–4287 (1996). 16535454 10.1128/aem.62.11.4284-4287.1996 PMC1388992

[b13] Kalishwaralal K., BarathManiKanth S., Pandian S. R., Deepak V. & Gurunathan S. Silver nanoparticles impede the biofilm formation by Pseudomonas aeruginosa and Staphylococcus epidermidis. Colloids Surf B Biointerfaces 79, 340–344 (2010). 20493674 10.1016/j.colsurfb.2010.04.014

[b14] Kumar C. G. & Mamidyala S. K. Extracellular synthesis of silver nanoparticles using culture supernatant of Pseudomonas aeruginosa. Colloids Surf B Biointerfaces 84, 462–466 (2011). 21353501 10.1016/j.colsurfb.2011.01.042

[b15] Taglietti A. *et al.* Antibiofilm activity of a monolayer of silver nanoparticles anchored to an amino-silanized glass surface. Biomaterials 35, 1779–1788 (2014). 24315574 10.1016/j.biomaterials.2013.11.047

[b16] Atiyeh B. S., Costagliola M., Hayek S. N. & Dibo S. A. Effect of silver on burn wound infection control and healing: review of the literature. Burns 33, 139–148 (2007). 17137719 10.1016/j.burns.2006.06.010

[b17] Mishra Y. *et al.* Synthesis and characterization of Ag nanoparticles in silica matrix by atom beam sputtering. Scripta Materialia 56, 629–632 (2007).

[b18] Mishra Y. *et al.* Synthesis and characterization of Ag-polymer nanocomposites. Journal of nanoscience and nanotechnology 10, 2833–2837 (2010). 20355509 10.1166/jnn.2010.1449

[b19] Singh P. *et al.* Biosynthesis, characterization, and antimicrobial applications of silver nanoparticles. Int J Nanomedicine 10, 2567–2577 (2015). 25848272 10.2147/IJN.S72313 PMC4386786

[b20] Mittal A. K. *et al.* Bio-synthesis of silver nanoparticles using Potentilla fulgens Wall. ex Hook. and its therapeutic evaluation as anticancer and antimicrobial agent. Mater Sci Eng C Mater Biol Appl 53, 120–127 (2015). 26042698 10.1016/j.msec.2015.04.038

[b21] Lee J. H., Kim Y. G., Cho M. H. & Lee J. ZnO nanoparticles inhibit Pseudomonas aeruginosa biofilm formation and virulence factor production. Microbiol Res 169, 888–896 (2014). 24958247 10.1016/j.micres.2014.05.005

[b22] Nafee N. *et al.* Antibiotic-free nanotherapeutics: ultra-small, mucus-penetrating solid lipid nanoparticles enhance the pulmonary delivery and anti-virulence efficacy of novel quorum sensing inhibitors. J Control Release 192, 131–140 (2014). 24997276 10.1016/j.jconrel.2014.06.055

[b23] Duran N., Marcato P. D., Alves O. L., Souza G. I. & Esposito E. Mechanistic aspects of biosynthesis of silver nanoparticles by several Fusarium oxysporum strains. J Nanobiotechnology 3, 8 (2005). 16014167 10.1186/1477-3155-3-8 PMC1180851

[b24] Verma V. C., Kharwar R. N. & Gange A. C. Biosynthesis of antimicrobial silver nanoparticles by the endophytic fungus Aspergillus clavatus. Nanomedicine (Lond) 5, 33–40 (2010). 20025462 10.2217/nnm.09.77

[b25] Dias M. A., Lacerda I. C., Pimentel P. F., de Castro H. F. & Rosa C. A. Removal of heavy metals by an Aspergillus terreus strain immobilized in a polyurethane matrix. Lett Appl Microbiol 34, 46–50 (2002). 11849492 10.1046/j.1472-765x.2002.01040.x

[b26] Musarrat J., Dwivedi S., Singh B. R., Al-Khedhairy A. A., Azam A. & Naqvi A. Production of antimicrobial silver nanoparticles in water extracts of the fungus Amylomyces rouxii strain KSU-09. Bioresour Technol 101, 8772–8776 (2010). 20619641 10.1016/j.biortech.2010.06.065

[b27] Singh B. N., Rawat A. K., Khan W., Naqvi A. H. & Singh B. R. Biosynthesis of stable antioxidant ZnO nanoparticles by Pseudomonas aeruginosa rhamnolipids. PLoS One 9, e106937 (2014). 25187953 10.1371/journal.pone.0106937 PMC4154833

[b28] Mulvaney P. Surface Plasmon Spectroscopy of Nanosized Metal Particles. Langmuir 12, 788–800 (1996).

[b29] Kumar M., Sandeep C. S. S., Kumar G., Mishra Y. K., Philip R. & Reddy G. B. Plasmonic and Nonlinear Optical Absorption Properties of Ag:ZrO2 Nanocomposite Thin Films. Plasmonics 9, 129–136 (2014).

[b30] Devi T. P., Kulanthaivel S., Kamil D., Borah J. L., Prabhakaran N. & Srinivasa N. Biosynthesis of silver nanoparticles from Trichoderma species. Indian J Exp Biol 51, 543–547 (2013). 23898553

[b31] Fuqua C., Parsek M. R. & Greenberg E. P. Regulation of gene expression by cell-to-cell communication: acyl-homoserine lactone quorum sensing. Annu Rev Genet 35, 439–468 (2001). 11700290 10.1146/annurev.genet.35.102401.090913

[b32] Kohler T., Curty L. K., Barja F., van Delden C. & Pechere J. C. Swarming of Pseudomonas aeruginosa is dependent on cell-to-cell signaling and requires flagella and pili. J Bacteriol 182, 5990–5996 (2000). 11029417 10.1128/jb.182.21.5990-5996.2000 PMC94731

[b33] Hoiby N. Diffuse panbronchiolitis and cystic fibrosis: East meets West. Thorax 49, 531–532 (1994). 8016786 10.1136/thx.49.6.531 PMC474936

[b34] Hentzer M. & Givskov M. Pharmacological inhibition of quorum sensing for the treatment of chronic bacterial infections. J Clin Invest 112, 1300–1307 (2003). 14597754 10.1172/JCI20074 PMC228474

[b35] Singh B. N. *et al.* Development and characterization of a novel Swarna-based herbo-metallic colloidal nano-formulation - inhibitor of Streptococcus mutans quorum sensing. RSC Advances 5, 5809–5822 (2015).

[b36] Naik K. & Kowshik M. Anti-quorum sensing activity of AgCl-TiO2 nanoparticles with potential use as active food packaging material. Journal of Applied Microbiology 117, 972–983 (2014). 24965598 10.1111/jam.12589

[b37] Hentzer M. *et al.* Attenuation of Pseudomonas aeruginosa virulence by quorum sensing inhibitors. EMBO J 22, 3803–3815 (2003). 12881415 10.1093/emboj/cdg366 PMC169039

[b38] Rasmussen T. B. *et al.* Screening for quorum-sensing inhibitors (QSI) by use of a novel genetic system, the QSI selector. J Bacteriol 187, 1799–1814 (2005). 15716452 10.1128/JB.187.5.1799-1814.2005 PMC1063990

[b39] Davies D. G., Parsek M. R., Pearson J. P., Iglewski B. H., Costerton J. W. & Greenberg E. P. The involvement of cell-to-cell signals in the development of a bacterial biofilm. Science 280, 295–298 (1998). 9535661 10.1126/science.280.5361.295

[b40] Rashid M. H. & Kornberg A. Inorganic polyphosphate is needed for swimming, swarming, and twitching motilities of Pseudomonas aeruginosa. Proc Natl Acad Sci USA 97, 4885–4890 (2000). 10758151 10.1073/pnas.060030097 PMC18327

[b41] Musthafa K. S., Sivamaruthi B. S., Pandian S. K. & Ravi A. V. Quorum sensing inhibition in Pseudomonas aeruginosa PAO1 by antagonistic compound phenylacetic acid. Curr Microbiol 65, 475–480 (2012). 22782469 10.1007/s00284-012-0181-9

[b42] Singh B. R., Singh B. N., Khan W., Singh H. B. & Naqvi A. H. ROS-mediated apoptotic cell death in prostate cancer LNCaP cells induced by biosurfactant stabilized CdS quantum dots. Biomaterials 33, 5753–5767 (2012). 22594971 10.1016/j.biomaterials.2012.04.045

[b43] Shoeb M. *et al.* ROS-dependent anticandidal activity of zinc oxide nanoparticles synthesized by using egg albumen as a biotemplate. Advances in Natural Sciences: Nanoscience and Nanotechnology 4, 035015 (2013).

[b44] Siddique Y. H. *et al.* Evaluation of the toxic potential of graphene copper nanocomposite (GCNC) in the third instar larvae of transgenic Drosophila melanogaster (hsp70-lacZ)Bg(9.). PLoS One 8, e80944 (2013). 24339891 10.1371/journal.pone.0080944 PMC3855226

[b45] Rao R. A. K., Singh S., Singh B. R., Khan W. & Naqvi A. H. Synthesis and characterization of surface modified graphene–zirconium oxide nanocomposite and its possible use for the removal of chlorophenol from aqueous solution. Journal of Environmental Chemical Engineering 2, 199–210 (2014).

[b46] Adonizio A. L., Downum K., Bennett B. C. & Mathee K. Anti-quorum sensing activity of medicinal plants in southern Florida. J Ethnopharmacol 105, 427–435 (2006). 16406418 10.1016/j.jep.2005.11.025

[b47] Singh B. N., Singh B. R., Singh R. L., Prakash D., Sarma B. K. & Singh H. B. Antioxidant and anti-quorum sensing activities of green pod of Acacia nilotica L. Food Chem Toxicol 47, 778–786 (2009). 19168114 10.1016/j.fct.2009.01.009

[b48] Shaw P. D. *et al.* Detecting and characterizing N-acyl-homoserine lactone signal molecules by thin-layer chromatography. Proc Natl Acad Sci USA 94, 6036–6041 (1997). 9177164 10.1073/pnas.94.12.6036 PMC20996

[b49] Spangenberg C., Winterpacht A., Zabel B. U. & Lobbert R. W. Cloning and characterization of a novel gene (TM7SF1) encoding a putative seven-pass transmembrane protein that is upregulated during kidney development. Genomics 48, 178–185 (1998). 9521871 10.1006/geno.1997.5170

