# Supplementary material for: Mycofabricated biosilver nanoparticles interrupt Pseudomonas aeruginosa quorum sensing systems
Source: Sci Rep. 2015 Sep 8;5:13719. doi: 10.1038/srep13719 (PMC4562228; doi:10.1038/srep13719)
Supplement: Supplementary Information [file srep13719-s1.pdf]

Supplementary Information

Mycofabricated biosilver nanoparticles interrupt *Pseudomonas aeruginosa* quorum sensing systems

Supplementary results

Identification of fungal strain. The isolated fungal strain was sub-cultured on potato dextrose agar (PDA) (Supplementary figure 1A) and in potato dextrose broth (PDB) media (Supplementary figure 1B). Light microscope images revealed the appearance of mycelia consisted of abundant sporangia resembling *Rhizopus* with small apophysis and erected sporangiophores without rhizoid formation (Supplementary figure 1C). Amplification and sequencing of internal transcribed spacer (ITS) region of fungal rDNA resulted in ~580bp long nucleotide sequence (Supplementary figure 1D inset). The blastn and pair-wise two sequence alignment analysis revealed ~99% sequence homology of ITS rDNA with *R. arrhizus* strains. Based on the phylogenetic analysis, the strain BRS-07 had been grouped in a cluster of *R. arrhizus* strains with high bootstrap values of 69–90%. The strain exhibited a close relationship with *R. arrhizus* strains including ATCC 24794, ATCC 11145, and ATCC 22580 and designated as *R. arrhizus* BRS-07 (Supplementary figure 1D).

Characterization of mfAgNPs. Thermal stability of the mfAgNPs was monitored by thermal gravimetric analysis (TGA) and depicted in Supplementary figure 3D. Obtained data show two step weight losses in the temperature region 50-750 ^{0}C. The initial weight loss examined was associated with the loss of adsorbed water molecules from protein matrix. However, the second weight loss can be due to the conversion of proteins and enzymes to carbon residue. Thermal degradation pattern was examined for the certain reductases and shuttle quinines of *Fusarium oxysporum* biomass in earlier study [1]. A less percent weight loss was also observed at 100-200 ^{0}C, while 50% weight loss was noticed at 300–450 ^{0}C. These findings reveal the better thermal stability of synthesized mfAgNPs.

Dose confirmation of mfAgNPs. First of all, a MIC assay was performed to discard any possible inhibitory effect of the mfAgNPs on PAO1 growth. It was confirmed that the bacterial growth was not inhibited during overnight treated with 25 µg/mL of mfAgNPs (data not shown). The impact of mfAgNPs on growth of PAO1 was further assessed using disc-diffusion assay. The zones of inhibition were recorded by 0, 19.2, and 30.1 mm, when bacterium exposed to 25, 50, and 100 µg/mL of mfAgNPs respectively (Supplementary figures 6Aa–d). However, sodium borohydride (SBH)-synthesized AgNPs at 25 µg/mL showed a strong inhibitory effect on the growth of PAO1 (Supplementary figure 6A-e). Non-toxic effect of 25 µg/mL of mfAgNPs was further confirmed using growth kinetic assay (Supplementary figure 6B). The overall observation confirms encapsulation of AgNPs by biomolecules of BAE of BRS-07, which could promote the sustained release of Ag^+ ions from mfAgNPs. The result is consistent with recent studies, proteins adsorption on the surface of NPs alters their biological effect [2].

Stability of mfAgNPs. Eventually, stability of the mfAgNPs was also determined at room temperature and in culture media by UV-Vis spectroscopy. No significant changes were observed in the absorbance at 410 nm up to 330 days of the storage at room temperature (Supplementary figure 11A). Higher stability of the mfAgNPs could be associated with the proteins-NPs interaction via free amine groups or cysteine residues, which leads to the encapsulation of NPs [3]. Similarly, higher stability was also observed, when 100 µg/mL of mfAgNPs added in nutrient broth (NB) culture media for 72 h (Supplementary figures 11B, C, and D) as compared to SBH-synthesized AgNPs (Supplementary figure 11E), which confirms the deposition of stabilizing agents on the surface of NPs.

Supplementary methods

Isolation and maintenance of fungal isolate. The roots of Ashawgandha (*Withania somnifera* L.) were obtained from the botanical garden of Banaras Hindu University, Varanasi, India (Longitude of 83.0 and the Latitude of 25.20). About 10 root fragments of 1.0 cm long and 3–5 mm wide were excavated from the soil (20–50 cm depth). Root fragments were washed thoroughly with sterilized DW and surface sterilized using 0.2% mercury chloride solution. After washing, fragments were dried on sterile blotting paper under aseptic conditions and placed on the PDA (pH 6.5 $\pm$ 0.2) medium. The plate was incubated at 27 $\pm$ 1 ^{0}C in complete darkness for 72 h.

Morphological and molecular characterization of fungal isolate. The presumptive identification of isolated fungus was done on the basis of spore morphological examination using methylene blue (HiMedia, India) staining technique. For molecular characterization, total genomic DNA was extracted from ~50 mg of mycelia using the QIAamp DNA Mini Kit (Qiagen, Inc., USA) according to the manufacturer's instructions. ITS region (ITS 1–5.8S–ITS 2) was amplified using primer set: ITS1-5′-TCCGTAGGTGAACCTGCGG-3′ and ITS4-5′-TCCTCCGCTTATTGATATGC-3′. Briefly, 50 ng of genomic DNA was used as template in 50 µL PCR mixture containing 0.20 mM dNTPs, 25 pmole of each primer, 5X *Taq* polymerase buffer (10 µL), 2 mM $MgCl_2$, and 0.2U *Taq* DNA polymerase. The PCR parameters consisted of 30 cycles of denaturation at 94 ^{0}C for 1 min, annealing at 50 ^{0}C for 1 min, and extension at 72 ^{0}C for 1 min with an initial denaturation. The final extension was performed at 94 ^{0}C and 72 ^{0}C for 3 and 7 min, respectively on Gene Amp PCR 9700 machine (Perkin Elmer Life and Analytical Sciences, CT, USA). The PCR product was analyzed on 1.0 % agarose gel containing ethidium bromide (10 mg/mL) (Sigma, USA) and documented on Universal Hood II-gel documentation

system (Bio-Rad, USA). The amplicons were sequenced using an automated ABI-Prism 377 DNA Sequencer (Applied Biosystems Inc., CA, USA).

Multiple sequence alignments and phylogenetic analysis. ITS sequences were compared with the fungal sequences retrieved from GenBank using the Blastn [4]. Multiple sequence analysis was performed using CLUSTALW with default parameters [5]. A phylogenetic tree was constructed by the neighbour-joining method with nucleotide pair-wise genetic distances and corrected by Kimura's two-parameter method using TreeCon tool software [6]. The reliability of tree topology was subjected to a bootstrap test and numbers at nodes indicate bootstrap support values as a percentage of 1000 replications. All branches with <50% bootstrap support were judged as inconclusive and collapsed. The branch lengths for all trees were normalized to 0.02% divergence.

Ag^+ ion release assay. Ag^+ ion release experiments were conducted in 250 mL glass media bottles containing 100 mL of NB medium. The medium has the following salts composition (g/L) shown in parentheses: peptone (5), meat extract (1), yeast extract (2), and NaCl (5) with the pH of medium was 7.0 ± 0.2. The mfAgNPs (25 µg/mL) was added to the medium, and then the glass media bottle was placed in dark with no shaking. Every 24 h, 10 mL of the medium was sampled from the bottle and centrifuged for 40 min at 5000g. Three microliters of culture supernatant was mixed with 2 mL of 65% nitric acid for inductively coupled plasma mass spectrometry (ICP-MS) analysis.

MTT assay. Cells were grown in 96-well microtiter plates for 4 h and treated with 25 µg/mL mfAgNPs for various time intervals 0, 12, 24, and 48 h. Hundred microliters of 3-[4,5-dimethylthiazol-2-yl]-2,5- diphenyltetrazolium bromide; thiazolyl blue (MTT; 0.5 mg/mL;

dissolved in culture medium) was added into each well and incubated at 30 ^{0}C for 3.5 h. After aspiration of medium, cells were incubated with 100 µL of acidic isopropanol (0.09 N HCl) for 15 min to dissolve the formazan crystals. The absorbance of MTT formazans was recorded at A_{570} nm using microplate reader (Bio-Rad, USA).

Determination of virulence factors production in PAO1. Cells were grown in AB minimal medium and prepared the supernatant for assessment of virulence factors as described by Hentzer *et al*. [7]. The activity of LasA protease was determined according to the method of Kong *et al*. [8]. Elastin congo red (ECR) was used as a substrate for the determination of LasB elastase activity using UV-vis spectrophotometer [9]. Briefly, 100 µL of bacterial supernatant was mixed with 900 µL of ECR buffer (100 mM Tris, 1.1 mM $CaCl_2$, 20 mg ECR and pH 7.2) and incubated at 37 ^{0}C under constant shaking for 3 h. Insoluble ECR was removed by centrifugation at 8000 rpm and recorded the absorbance of filtrate at A_{495} nm. Content of pyocyanin was determined using the solvent system of chloroform-methanol (3:2), re-extracted with 1.0 mL of 0.2N HCl, and measured the absorbance at A_{540} nm [10]. Production of pyoverdin was quantified as described by Cox and Adams [11]. For quantification of pyochelin, 1 mL of culture filtrate, 1 mL of 1N NaOH, nitrite molybdate reagent, and 1 mL of 0.5N HCl were mixed together. The final volume of reaction mixture was made up to 5 mL by adding DW and measured the absorbance at A_{510} nm.

Rhamnolipid production was determined by adjusting the pH of culture supernatant with concentrated HCl, centrifuged at 10,000 rpm for 5 min, and eventually recorded the absorbance at A_{570} nm [12]. The contents of extracellular and cell bounded rhamnolipids were also determined by TLC [13]. Briefly, 3 µL of extract (10 mg/mL) was spotted onto silica gel 60 F_{254} TLC plates (Merck, Germany) and the separation of rhamnolipids was achieved using a mobile phase of

chloroform-methanol-acetic acid (8:1.8:0.2). Subsequently, plates were stained with 1.6% of orcinol reagent (prepared in 15% of H_2SO_4) and incubated for 5 min at 100 ^{0}C.

In alginate production assay, cells were cultured in LB broth medium (10 mL) and incubated for 3 days at 30 ^{0}C under the stationary phase condition to form biofilm. The exhausted culture medium was collected and centrifuged at 8000 rpm for 10 min to remove the cells. Alginate content was precipitated by adding 2% of cetylpyridinium chloride and quantified using carbazole reagent [14].

Determination of rhamnolipid production using plate assay. Briefly, PAO1 culture was grown in the presence or absence of mfAgNPs and inoculated into NA medium containing 1 mM $MgSO_4$, 0.5 % tryptone, 0.02% cetyltrimethylammonium bromide (CTAB), methylene blue (0.0005%), and 1.5% agar and then plates were incubated at 30 ^{0}C for 48 h. The diameter of yellowish-green zone around the discs was considered for rhamnolipid production [13].

Swimming and swarming motility assay. For swimming assay, 10 µL overnight culture of PAO1 (10^6 cells/mL) was point inoculated at the center of NB medium containing 0.3% agar and different concentrations of mfAgNPs. Swarming assay was conducted as described in swimming assay instate of 0.3% agar, we used 0.5% agar. The plates were then incubated at 30 ^{0}C in upright position for 48 h. The reduction in swimming and swarming migrations was recorded by examining the swim and swarm zones of PAO1 cells.

Polyvinyl chloride (PVC) biofilm formation assay. Briefly, 200 µL culture of PAO1 (0.5 OD at A_{600} nm) was seeded in 96-well microtiter plates and exposed to different concentrations of mfAgNPs. After incubation of 24 h at 30 ^{0}C, plate was rinsed to remove planktonic cells and biofilms were stained with 0.25% crystal violet (CV) solution. After drying at room temperature,

biofilm formation was analyzed by light microscopy and photographed. For quantitative analysis, ethanol was used to solubilize CV and recorded the absorbance at A_{650} nm (Bio-Rad, USA) [15].

SEM analysis. Exponentially growing cells of PAO1 which had been exposed to mfAgNPs, washed twice with PBS, and fixed in 2.0% glutaraldehyde (prepared in 0.1 M phosphate buffer; pH 7.0). Treated cells were stained with saturated solutions of uranyl acetate and lead citrate. The samples were examined at 5 µm scale by TEM (JEM2100, JEOL, Japan) with an accelerating voltage of 10 kV.

Protein assay. Protein concentrations were determined by the Bio-Rad Protein Assay (Bio-Rad, USA). Raw data from all the assays were normalized to total protein concentration; however no significant difference between sample sets was observed.

Supplementary figure(s)

Supplementary figure 1

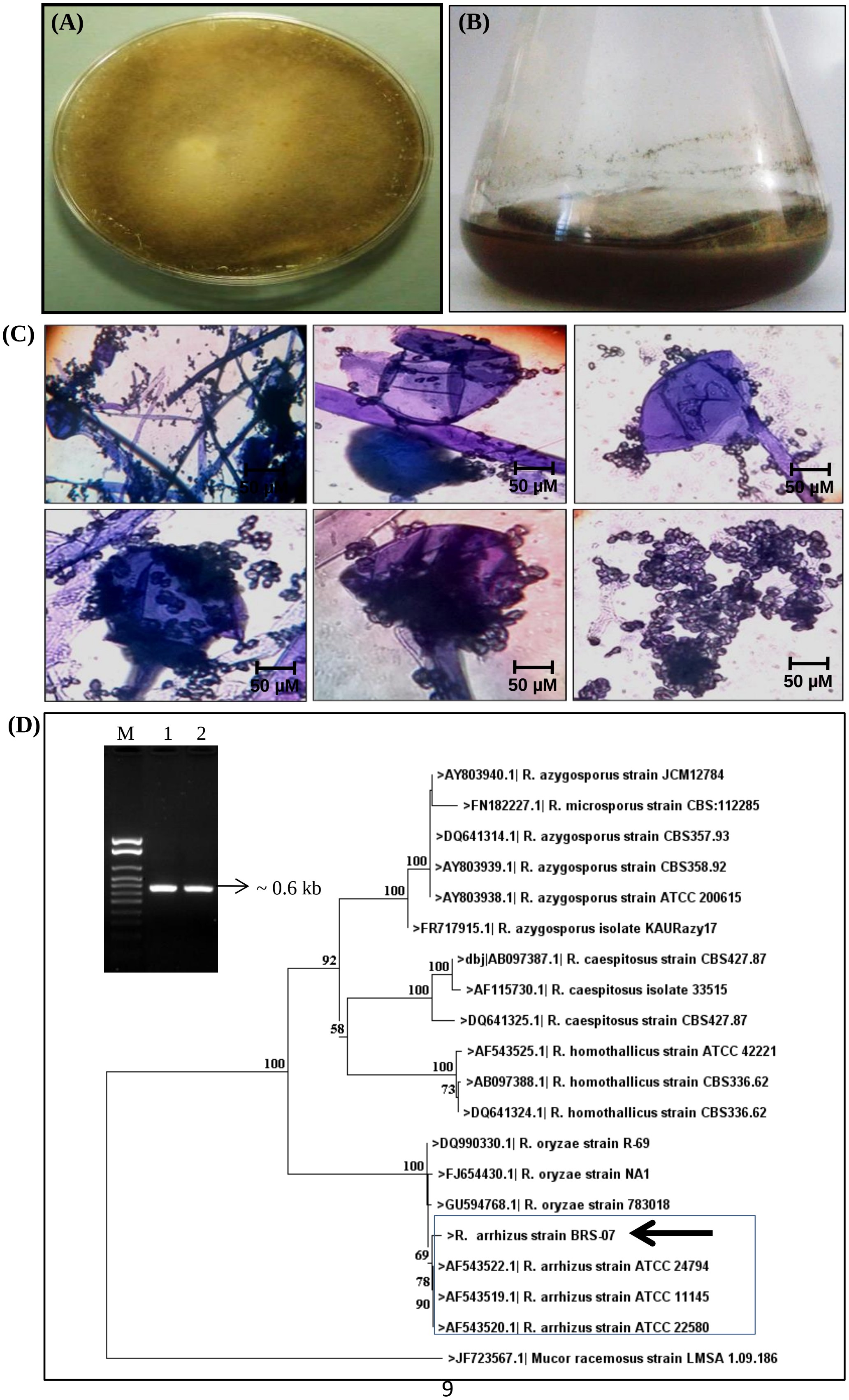

Supplementary figure 1 Molecular characterization of *R*. *arrhizus* BRS-07. Growth patterns of BRS-07 on (A) PDA and (B) PDB culture medium. (C) Light microscopic images show spores morphology of BRS-07 and their arrangement after staining with methylene blue. (D) Phylogenetic analysis shows relationship between BRS-07 and ITS sequences of other fungal strains. The bootstrap values of 50% or greater are indicated on the tree and arrow represents the status of *R*. *arrhizus* BRS-07 strain in phylogenetic tree. The scale bar indicates the numbers of nucleotide substitutions per site. Left corner, the inset shows the 1.2% agarose gel electrophoresis of PCR amplified ITS regions of BRS-07 rRNA. M; 100bp ladder molecular weight marker, lanes 1 and 2; ITS amplicons of *R. arrhizus* rRNA.

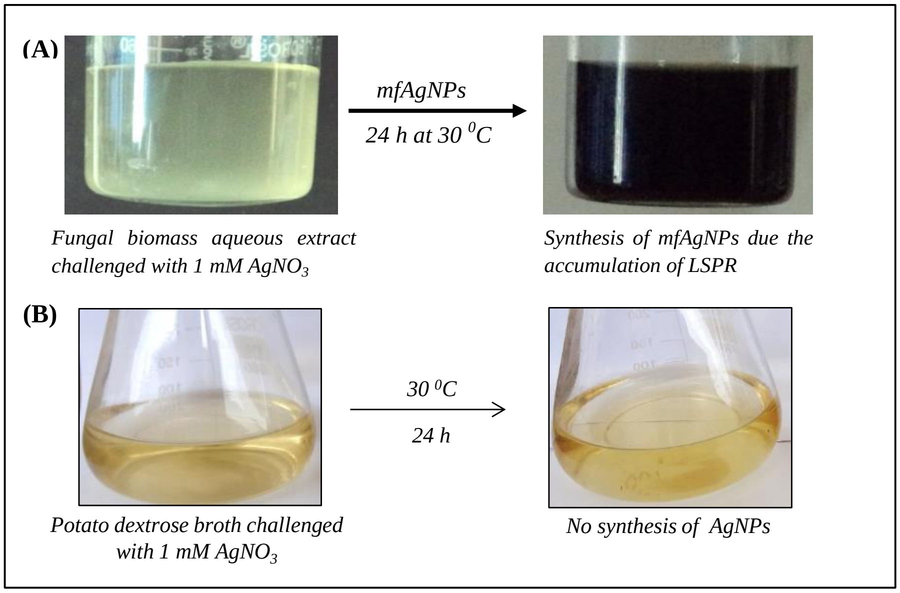

Supplementary figure 2 Biosynthesis of mfAgNPs. (A) Biomass aqueous extract of BRS-07 was challenged with 1 mM $AgNO_3$ solution for 24 h and (B) culture medium was used as a control.

Supplementary figure 3

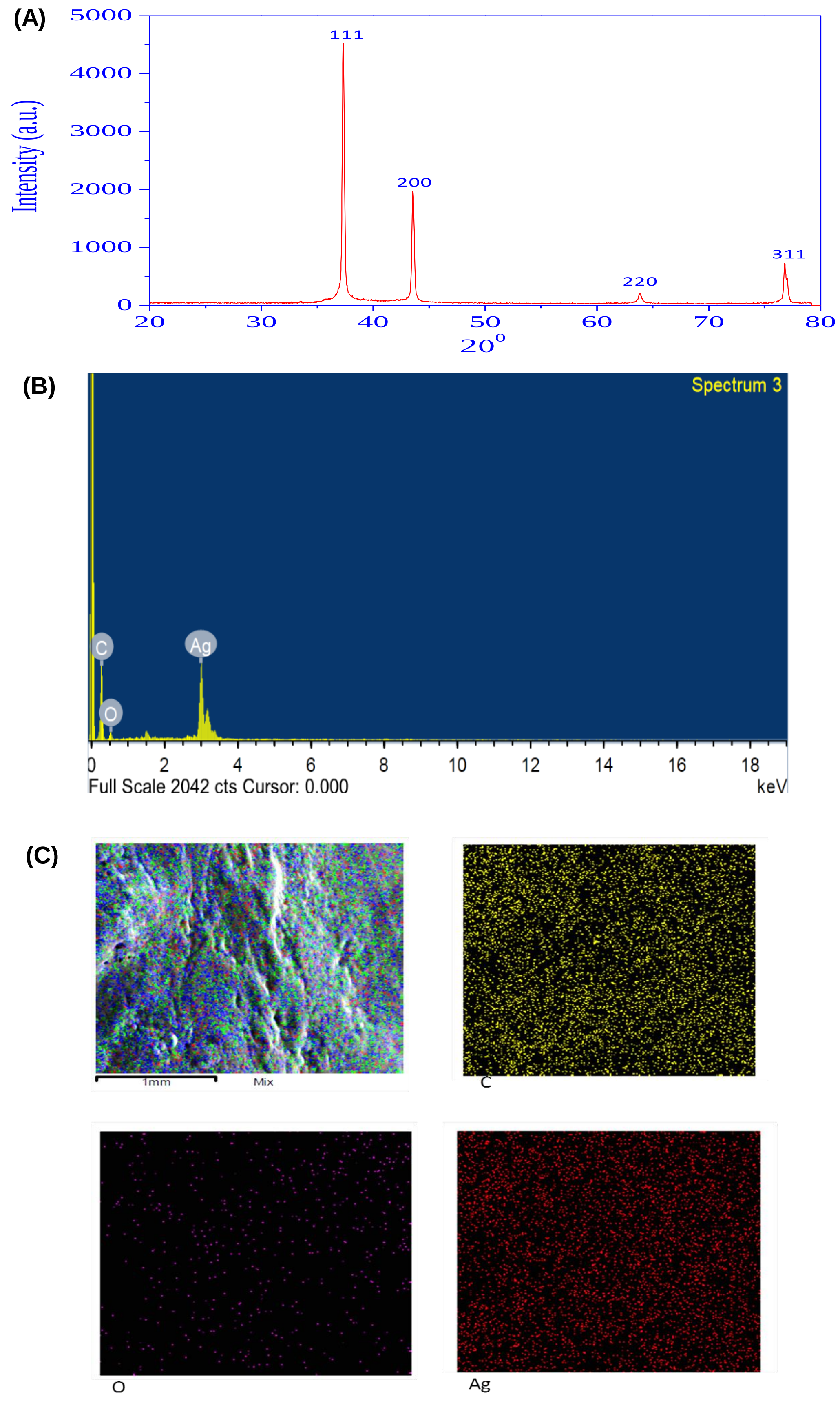

Supplementary figure 3

(D)

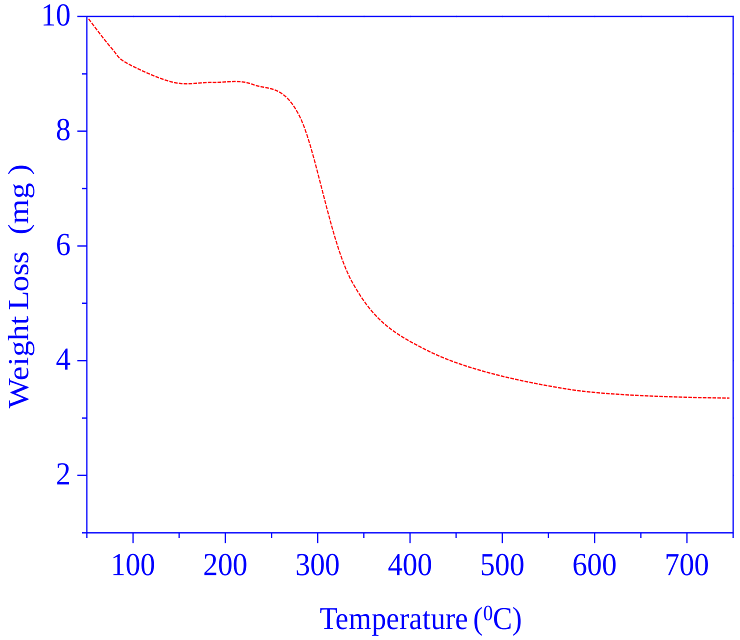

Supplementary figure 3 Characterization of mfAgNPs. (A) XRD patterns of mfAgNPs fabricated using BAE of BRS-07. (B) Elemental characterization of mfAgNPs. (B) EDAX analysis showing Ag peak. (C) Elemental mapping. (D) TGA spectrum.

Supplementary figure 4

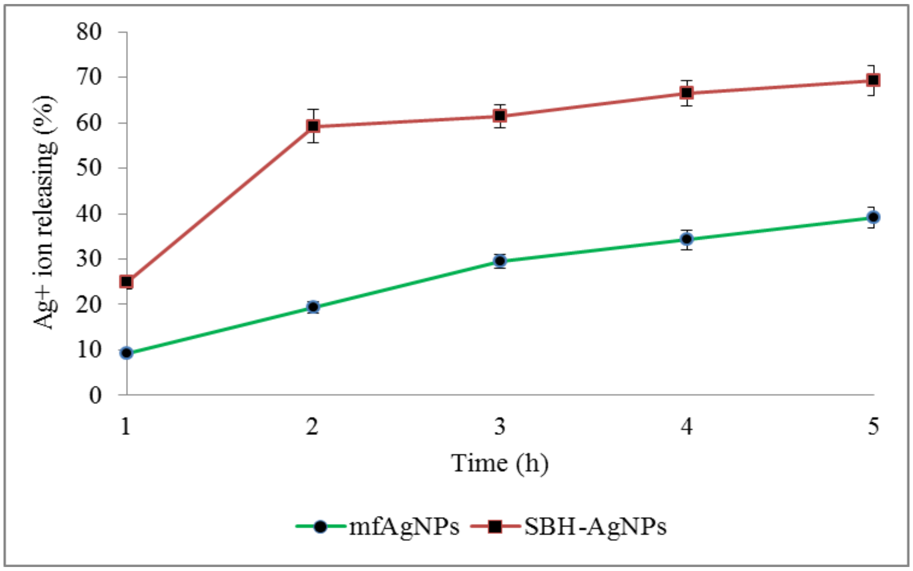

Supplementary figure 4 Time dependent Ag^{+} ions release from mfAgNPs and SHB-synthesized AgNPs in nutrient broth (NB) culture media. Each reported value represents the mean ± SD from three independent experiments.

Supplementary figure 5

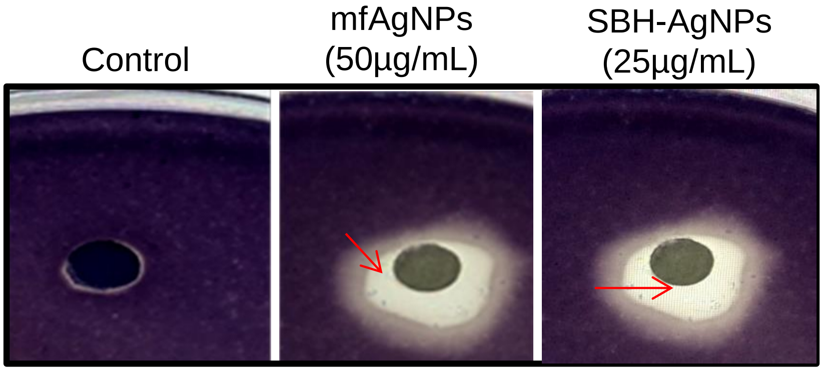

Supplementary figure 5 Assessment of antibacterial activity. *C. violaceum* 12472 was treated with 50 µg/mL of mfAgNPs and 25 µg/mL of SBH-synthesized AgNPs for 24 h. A clear and transparent zone was observed (red arrows) which indicate cidal effect of test materials.

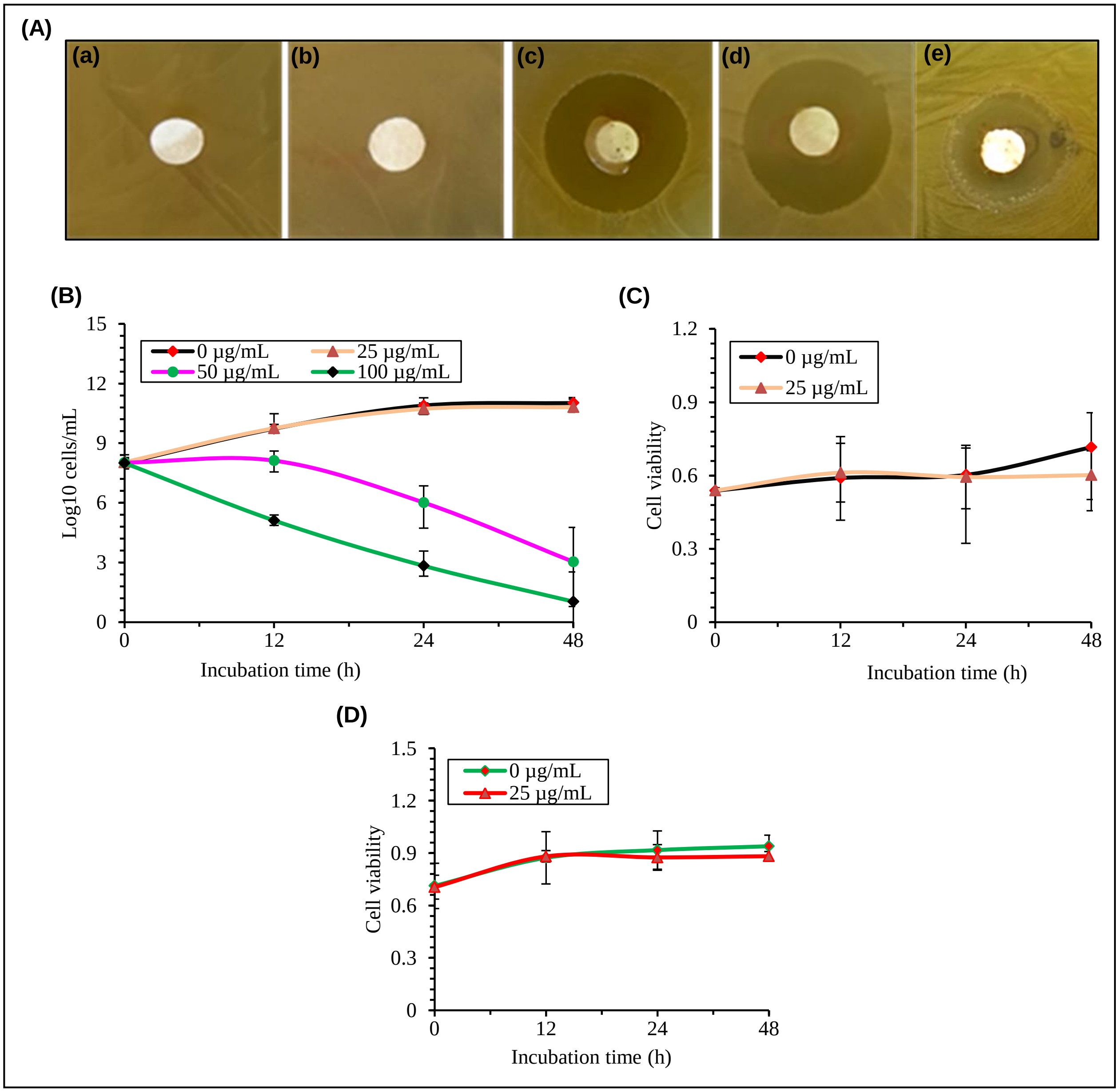

Supplementary figure 6 Effect of mfAgNPs on cell viability. (A) A disc diffusion method was used for detection of anti-bacterial activity. For this, overnight log phase grown PAO1 culture (100 µL) spread on LB agar plate and added different concentration of the mfAgNPs (a) 0 µg/mL (b) 25 µg/mL, (c) 50 µg/mL, (d) 100 µg/mL and (e) SBH synthesized AgNPs (25 µg/mL) on sterile paper discs. The plates were incubated overnight at 30 ^{0}C for 24 h. At 25 µg/mL, mfAgNPs show non-cidal effect, while 50 and 100 µg/mL of mfAgNPs as well as SHB synthesized AgNPs at 25 µg/mL exhibit bactericidal effects. (B) Cells were grown in the presence of different concentrations of mfAgNPs at 30 ^{0}C and counted at different time intervals using serial dilution plat technique. No mfAgNPs was supplemented in the control group. (C) Human prostate epithelial and (D) PAO1 cells were grown in 96-well plate for indicated time intervals in the absence or presence of 25 µg/mL mfAgNPs and determined cell viability by the MTT assay. The absorbance was measured at A_{450} nm with λ correction at 650 nm. Each reported value represents the mean ± SD from six independent experiments.

Supplementary figure 7

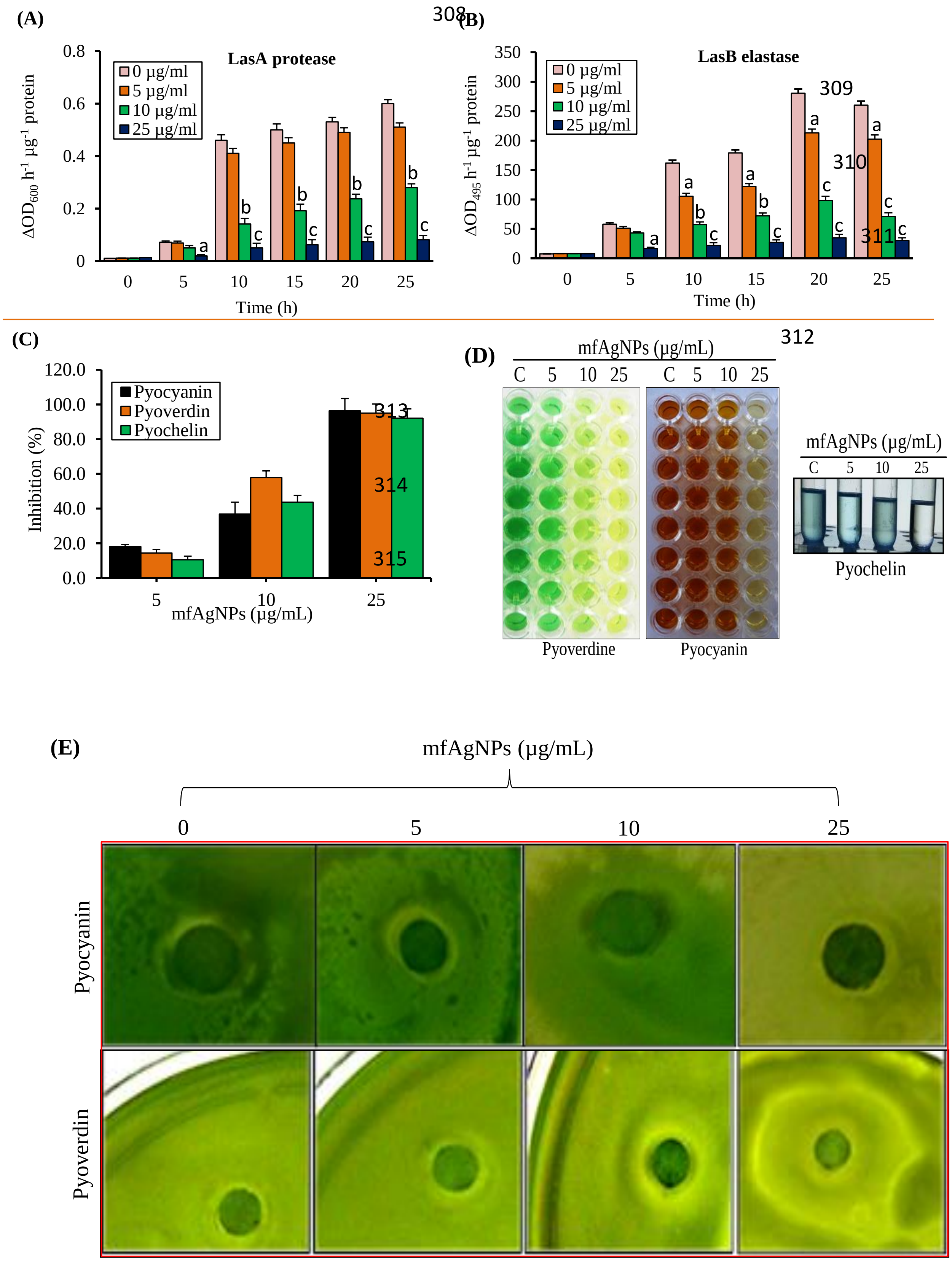

Supplementary figure 7

(F)

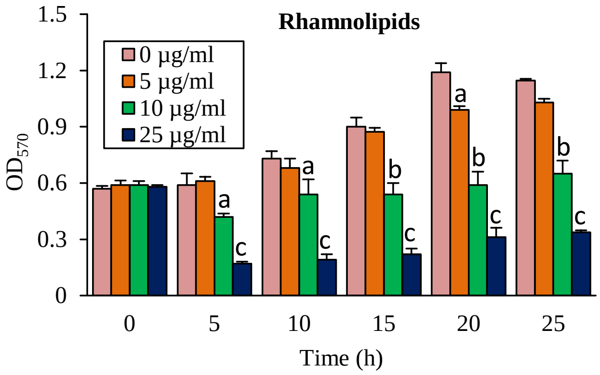

(G)

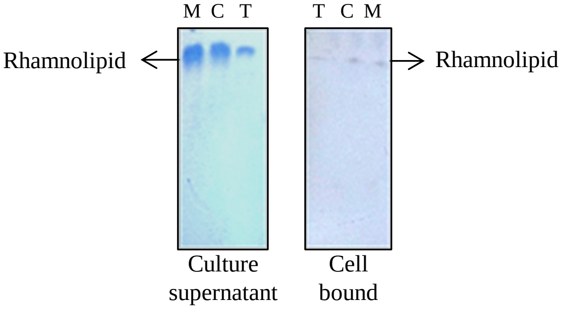

(H)

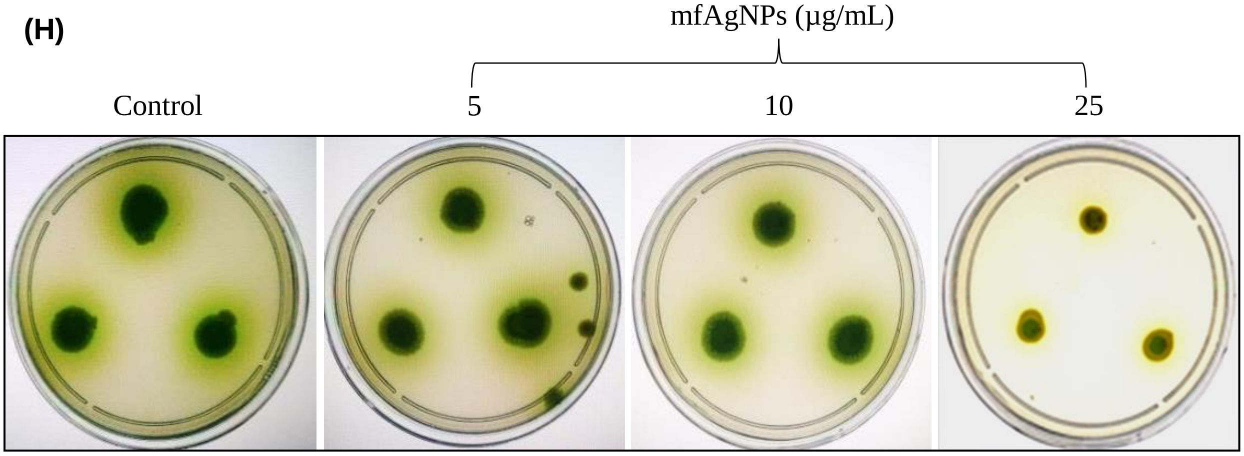

(I)

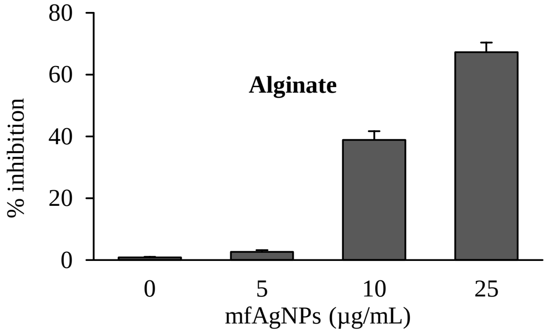

Supplementary figure 7 Effect of mfAgNPs on the secretion of PAO1 virulence factors. Production of virulence factors at different mfAgNPs concentrations were analyzed spectrophotometrically. (A) LasA protease activity. (B) LasB elastase activity. (C and D) siderophores content (pyocyanin, pyoverdin and pyochelin). (E) Pyocyanin and pyoverdin contents by agar plate method. (F) rhamnolipid content. (G) Rhamnolipid contents were also examined in culture filtrate and cell bound using TLC technique and plates were stained with a reagent containing orcinol and sulfuric acid in ethanol. M, standard rhamnose; C, untreated control; T, mfAgNPs (25 µg/mL). (I) Alginate content. Error bars indicate the SD of 3 measurements. c, P < 0.001 Vs control. b, P < 0.01 Vs the control. a, *P* < 0.05 Vs control.

Supplementary figure 8

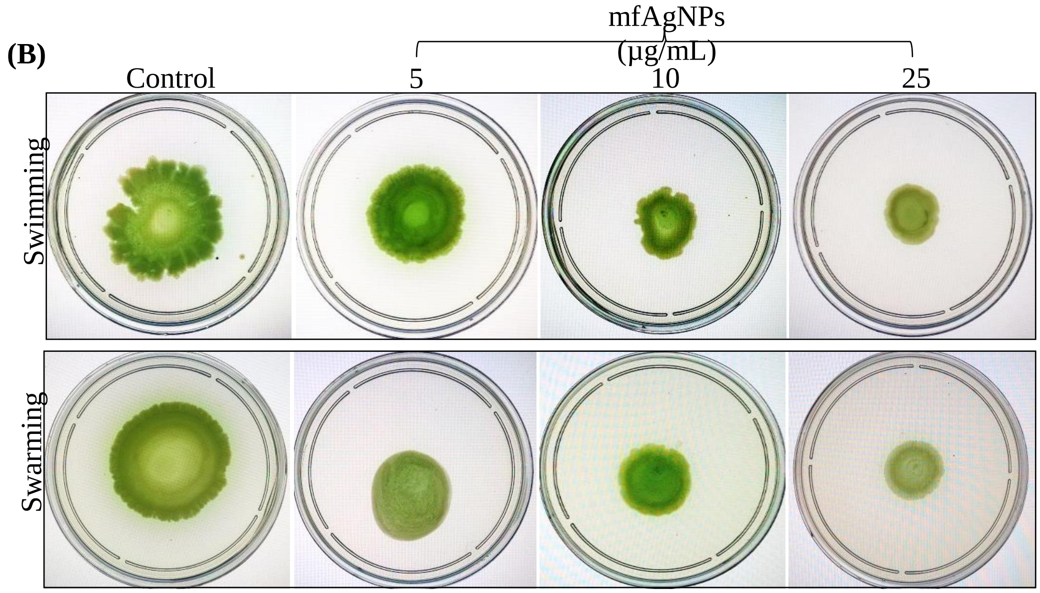

Supplementary figure 8 (A) Effect of mfAgNPs on rhamnolipid production. PAO1 was grown overnight in presence of indicated concentrations of mfAgNPs. Ten microliters culture from each treatment was inoculated on NA medium supplemented with $MgSO_4$, tryptone, CTAB, and methylene blue for 48 h at 37 ^{0}C. The diameter of yellowish-green zone around the discs was examined for the rhamnolipids production. (B) Impact of mfAgNPs on swimming–swarming motility and alginate production in PAO1. NB containing 0.3% and 0.5% agar was prepared for swimming and swarming motility, respectively. The mfAgNPs supplemented plates were point inoculated with PAO1. After 48 h of incubation, the inhibition of swimming and swarming motility was examined by measuring the diameters of PAO1 cells.

Supplementary figure 9

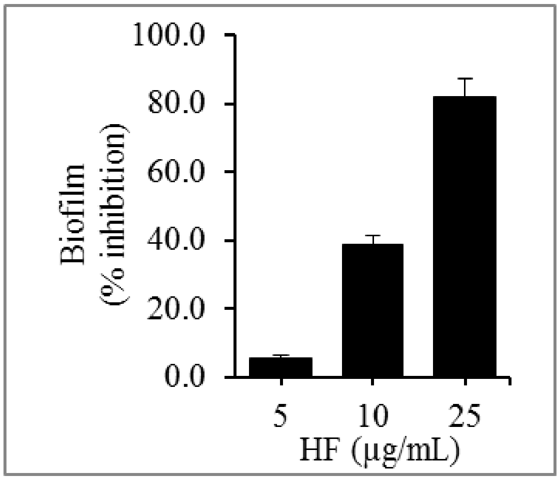

Supplementary figure 9 Concentration dependent anti-biofilm activity of HF at incubation of 48 h.

Supplementary figure 10

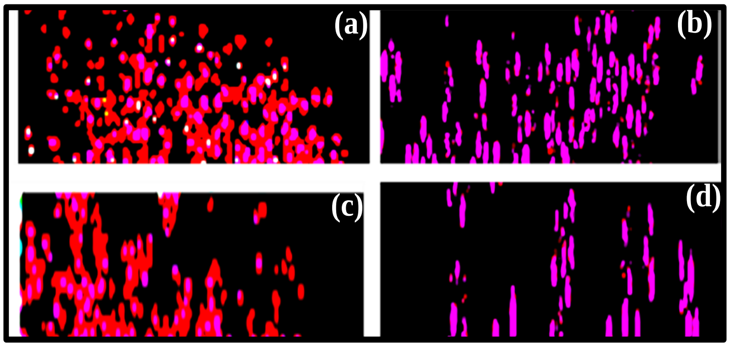

Supplementary figure 10 Effect of SDS-treatment of PAO1 biofilms. Matured biofilms (3 day old) were grown in the absence or presence of mfAgNPs (25 µg/mL) and treated with 0.1% SDS for 16 h. Bacterial biofilms were strained using SYTO-9 and PI fluorescent dies and examined by SLCM. Treatments include: (a) untreated control (b) 25 µg/mL mfAgNPs, (c) 0.1% SDS, and (d) 25 µg/mL mfAgNPS plus 0.1% SDS.

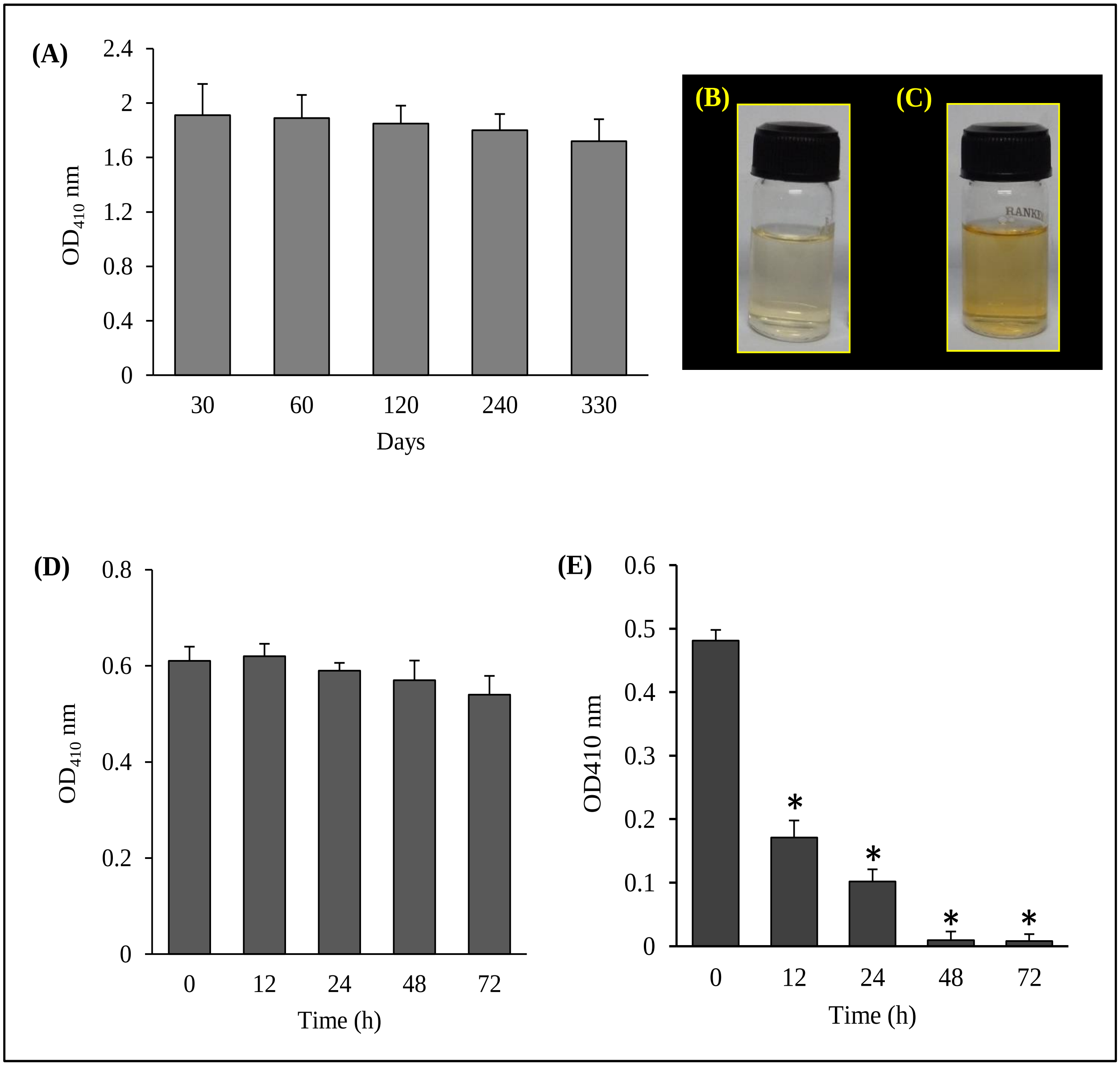

Supplementary figure 11 Stability of mfAgNPs. (A) Showing the stabilization during the storage up to 330 days. The time dependent absorbance data revealed that no significant changes in the absorbance at A_{410} nm wavelength in the aqueous state. (B and C) LB broth culture medium incubated without (B) and with 25 µg/mL of mfAgNPs (C) up to 72 h and exhibiting the well disparity without agglomeration. (D and E) Bar graphs show the time dependent change in absorbance A_{410} nm of mfAgNPs (D) and SBH-synthesized AgNPs (E) upon the incubation with LB broth culture medium for indicated time. Each reported value represents the mean ± SD from six independent experiments. *Significant at $P < 0.001$.

References

1. Duran N, Marcato PD, Alves OL, Souza GI, Esposito E. Mechanistic aspects of biosynthesis of silver nanoparticles by several Fusarium oxysporum strains. *J Nanobiotechnology* **3**, 8 (2005).

2. Pino Pd, Pelaz B, Zhang Q, Maffre P, Nienhaus GU, Parak WJ. Protein corona formation around nanoparticles - from the past to the future. *Materials Horizons* **1**, 301-313 (2014).

3. Sanghi R, Verma P. Biomimetic synthesis and characterisation of protein capped silver nanoparticles. *Bioresource Technology* **100**, 501-504 (2009).

4. Altschul SF*, et al.* Gapped BLAST and PSI-BLAST: a new generation of protein database search programs. *Nucleic Acids Res* **25**, 3389-3402 (1997).

5. Thompson JD, Higgins DG, Gibson TJ. CLUSTAL W: improving the sensitivity of progressive multiple sequence alignment through sequence weighting, position-specific gap penalties and weight matrix choice. *Nucleic Acids Res* **22**, 4673-4680 (1994).

6. Kimura M. A simple method for estimating evolutionary rates of base substitutions through comparative studies of nucleotide sequences. *J Mol Evol* **16**, 111-120 (1980).

7. Hentzer M*, et al.* Inhibition of quorum sensing in Pseudomonas aeruginosa biofilm bacteria by a halogenated furanone compound. *Microbiology* **148**, 87-102 (2002).

8. Kong KF*, et al.* Pseudomonas aeruginosa AmpR is a global transcriptional factor that regulates expression of AmpC and PoxB beta-lactamases, proteases, quorum sensing, and other virulence factors. *Antimicrob Agents Chemother* **49**, 4567-4575 (2005).

9. Ohman DE, Cryz SJ, Iglewski BH. Isolation and characterization of Pseudomonas aeruginosa PAO mutant that produces altered elastase. *J Bacteriol* **142**, 836-842 (1980).

10. Vandeputte OM, *et al.* Identification of catechin as one of the flavonoids from Combretum albiflorum bark extract that reduces the production of quorum-sensing-controlled virulence factors in Pseudomonas aeruginosa PAO1. *Appl Environ Microbiol* **76**, 243-253 (2010).

11. Cox CD, Adams P. Siderophore activity of pyoverdin for Pseudomonas aeruginosa. *Infect Immun* **48**, 130-138 (1985).

12. McClure CD, Schiller NL. Effects of Pseudomonas aeruginosa rhamnolipids on human monocyte-derived macrophages. *J Leukoc Biol* **51**, 97-102 (1992).

13. Caiazza NC, Shanks RM, O'Toole GA. Rhamnolipids modulate swarming motility patterns of Pseudomonas aeruginosa. *J Bacteriol* **187**, 7351-7361 (2005).

14. Franklin MJ, Ohman DE. Identification of algF in the alginate biosynthetic gene cluster of Pseudomonas aeruginosa which is required for alginate acetylation. *J Bacteriol* **175**, 5057-5065 (1993).

15. O'Toole GA, Kolter R. Flagellar and twitching motility are necessary for Pseudomonas aeruginosa biofilm development. *Mol Microbiol* **30**, 295-304 (1998).
